# Single-Crystal-to-Single-Crystal Cluster Transformation in a Microporous Molybdoarsenate(V)-Metalorganic Framework

**DOI:** 10.1021/acs.inorgchem.1c02276

**Published:** 2021-09-21

**Authors:** Nour Dissem, Beñat Artetxe, Leire San Felices, Garikoitz Beobide, Oscar Castillo, Estibaliz Ruiz-Bilbao, Luis Lezama, María dM. Vivanco, Amor Haddad, Juan M. Gutiérrez-Zorrilla

**Affiliations:** † Laboratoire de Matériaux, Cristallochimie et Thermodynamique Appliquée, Faculté des Sciences de Tunis, Université de Tunis El Manar, 2092 Tunis, Tunisia; ‡ Departamento de Química Inorgánica, Facultad de Ciencia y Tecnología, Universidad del País Vasco UPV/EHU, P.O. Box 644, 48080 Bilbao, Spain; § Servicios Generales de Investigación SGIker, Facultad de Ciencia y Tecnología, Universidad del País Vasco UPV/EHU, P.O. Box 644, 48080 Bilbao, Spain; ⊥ Laboratoire des Matériaux et Cristallochimie, Institut Supérieur des Sciences Appliquées et Technologie, 5111 Mahdia, Tunisia; + Cancer Heterogeneity Lab, Center for Cooperative Research in Biosciences (CIC bioGUNE), Basque Research and Technology Alliance (BRTA), Bizkaia Technology Park, 48160 Derio, Spain; ∥ BCMaterials, Basque Center for Materials Applications and Nanostructures, UPV/EHU Science Park, 48940 Leioa, Spain

## Abstract

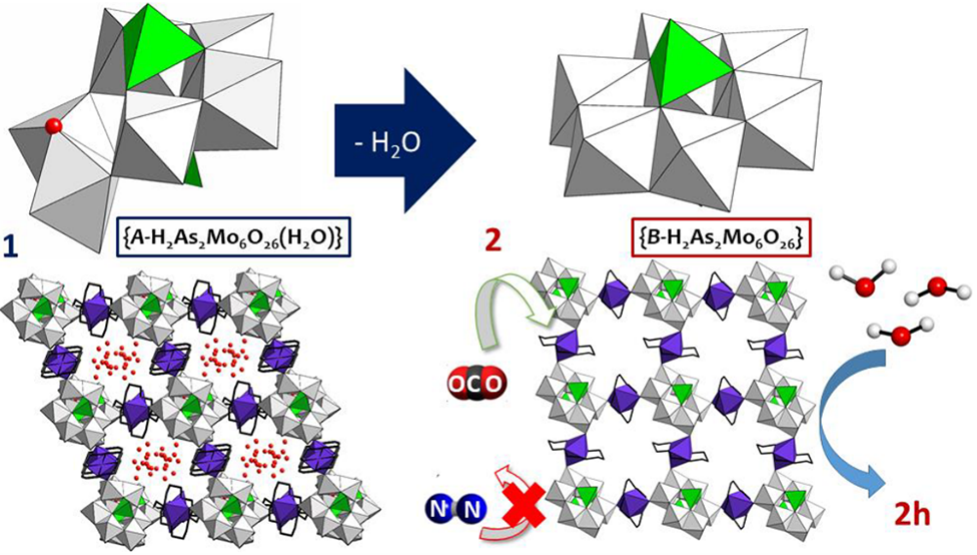

The hybrid compound [Cu(cyclam)(H_2_O)_2_]_0.5_[{Cu(cyclam)}_1.5_{*B*-H_2_As_2_Mo_6_O_26_(H_2_O)}]·9H_2_O (**1**) (cyclam = 1,4,8,11-tetraazacyclotetradecane) was synthesized in aqueous solution by reacting the {Cu(cyclam)}^2+^ complex with a mixture of heptamolybdate and an arsenate(V) source. Crystal packing of **1** exhibits a supramolecular open-framework built of discrete covalent molybdoarsenate/metalorganic units and additional [Cu(cyclam)(H_2_O)_2_]^2+^ cations, the stacking of which generates squarelike channels parallel to the *z* axis with an approximate cross section of 10 × 11 Å^2^ where all the hydration water molecules are hosted. Thermal evacuation of solvent molecules yields a new anhydrous crystalline phase, but compound **1** does not preserve its single-crystalline nature upon heating. However, when crystals are dehydrated under vacuum, they undergo a structural transformation that proceeds via a single-crystal-to-single-crystal pathway, leading to the anhydrous phase [{Cu(cyclam)}_2_(*A*-H_2_As_2_Mo_6_O_26_)] (**2**). Total dehydration results in important modifications within the inorganic cluster skeleton which reveals an unprecedented solid-state *B* to *A* isomerization of the polyoxoanion. This transition also involves changes in the Cu^II^ bonding scheme that lead to covalent cluster/metalorganic layers by retaining the open-framework nature of **1**. Compound **2** adsorbs ambient moisture upon air exposure, but it does not revert back to **1**, and the hydrated phase [{Cu(cyclam)}_2_(*A*-H_2_As_2_Mo_6_O_26_)]·6H_2_O (**2h**) is obtained instead. Structural variations between **1** and **2** are reflected in electron paramagnetic resonance spectroscopy measurements, and the permanent microporosity of **2** provides interesting functionalities to the system such as the selective adsorption of gaseous CO_2_ over N_2_.

## Abstract 2

A supramolecular open framework built of copper(II)-complexes of a macrocyclic tetraaza ligand and arsenomolybdate anions undergoes a single-crystal-to-single-crystal transformation upon dehydration that implies an unprecedented solid state cluster isomerization. The permanent porosity of the system allows the selective adsorption of CO_2_ over N_2_.

## Introduction

The construction of polyoxometalate (POM)-based open-frameworks allows the combination of the intrinsic properties of these nanometric metal-oxo clusters (e.g., redox properties, magnetism, luminescence...)^1−3^ with the inherent features of porous materials, such as high internal surface area.^4,5^ Thus, these systems represent promising candidates for applications in fields like gas sorption and separation, ion-exchange, sensing, and catalysis.^6^ Beyond the ionic crystals in which the nonefficient packing between macroanions and bulky [M_3_O(OOCR)_6_(L)_3_]^+^ cations generates large structural voids,^7,8^ different synthetic strategies have been followed to prepare extended POM-based porous solids. The direct linkage of cluster units through additional metal cations can lead to fully inorganic porous architectures with high thermal and chemical stabilities.^9,10^ However, this procedure usually lacks predictability, and hence, it avoids a previous rational design. Another elegant strategy involves the preparation of structural analogues of the well-known metalorganic frameworks (MOFs) by either connection of metal polysubstituted POMs through bridging organic ligands or the combination between metallic nodes and organically derivatized POMs that act as linkers.^11^ The multistep synthetic work required can be identified as a major drawback for this approach.

A simpler route implies the assembly of polyoxoanions with transition metal complexes. In this regard, some of us recently explored the use of copper(II) complexes of macrocyclic polyamines, because multidentate ligands can block the equatorial positions of the metal center and leave axial coordination sites available to connect contiguous POM units. Our latest studies afforded the multifunctional [Cu(cyclam)][{Cu(cyclam)}_2_(V_10_O_28_)]·10H_2_O (cyclam = 1,4,8,11-tetraazacyclotetradecane) supramolecular framework,^12^ which is able to selectively adsorb gaseous CO_2_ over N_2_ and exhibits catalytic activity toward the heterogeneous oxidation of adamantane as well as the covalent [{Cu(cyclam)}_3_(W_7_O_24_)]·15.5H_2_O hybrid with gas sorption ability.^13^ Furthermore, thermal activation of the former example did not alter the structure of the parent robust framework, but the latter system displayed interesting crystal dynamics which proceeded via single-crystal-to-single-crystal (SCSC) transformations.

Solid-phase transitions triggered by an external stimuli (e.g., heat, light, presence/absence of chemical species) in which single-crystallinity is retained constitute a very promising research field, because they allow the monitoring of how the position of atoms and molecules is modified all along the transformation. Moreover, the response offered by these smart systems could modify a given property from the parent material (e.g., luminescent, magnetic, sorptive). Thus, the rational control of the structure–properties relationship facilitates the fabrication of practical devices such as molecular switches, sensors, or data storage systems.^14,15^

To date, several SCSC transformations have been published for coordination compounds such as MOFs,^16,17^ but comparatively, there are only a few reports for POM-based systems.^18,19^ Our latest studies identified the {Cu(cyclam)}^2+^/POM family as a suitable candidate to prepare hybrid networks able to undergo SCSC transformations because the plasticity of copper(II) centers and the ability of macrocyclic ligands to establish cooperative supramolecular interactions with oxygen-rich POM surfaces avoids the loss of crystallinity along the phase transition. The thermal evacuation of guest solvent molecules usually triggers the occurrence of relevant structural changes such as the rearrangement of the Cu–O bonding scheme.^20^ However, no major skeletal modification of the metal-oxo anion was observed so far, besides the isolation of coordinatively unsaturated 3d-^21^ or 4f-metal^22^ substituted clusters such as {α-SiW_11_O_39_Cu} and {(α-GeW_11_O_39_)Ln(OAc)}_2_, respectively.

Despite their scarcity, solid-state transformations of POM clusters have long been known, as exemplified by the isomerization of the trilacunary [*A*-α-PW_9_O_34_]^9–^ Keggin–type anion into the *B* form upon heating.^23^ Among this class of phase transitions, there are only a few examples in which both the initial and final stages were structurally characterized by single-crystal X-ray diffraction. These include (i) the ring-opening polymerization of [V_4_O_12_]^4–^ cyclotetravanadate species which leads to metavanadate chains;^24^ (ii) thermal isomerization of the metastable [{Zn_2_(H_2_O)(OH)}_2_{Zn(H_2_O)_2_}(γ-HSiW_10_O_36_)_2_]^8–^ anion that results in two different sandwich-type POMs formed by either α- or β-Keggin-type trilacunary units and comprise the {Zn_2_W(O)O_3_}_2_^4+^ core;^25^ and (iii) temperature-triggered reversible transformation of cubane-type tetracobalt core sandwiched by POM units, into a planar rhomblike moiety in the [{Co(H_2_O)}_2_(OH)_2_{Co(H_2_O)_2_}_2_(H_2_SiW_10_O_36_)_2_]^8–^ species.^26^ Up until now, there is only one report in the literature in which such a kind of structural transformation proceeds via SCSC. In this case, the tetrabutylammonium salt of the [γ-SiV_2_W_10_O_39_]^4–^ anion displays a linear (μ-oxo)-divanadium(V) core which reacts with water to lead to the bis(μ-hydroxo)-divanadium(V) unit.^27^

In the course of our work on the {Cu(cyclam)}^2+^/molybdate system,^28^ we decided to extend these studies to heteropolymolybdates and more specifically to the As^V^/Mo combination. Beyond Keggin or Wells–Dawson type molybdoarsenates,^29,30^ we focused our attention on the smaller [H_2_As_2_Mo_6_O_26_]^4–^.^31−34^ Here we report on the hybrid [Cu(cyclam)(H_2_O)_2_]_0.5_[{Cu(cyclam)}_1.5_{*B*-H_2_As_2_Mo_6_O_26_(H_2_O)}]·9H_2_O (**1**) framework, which is able to undergo a nonreversible SCSC transformation induced by the evacuation of guest solvent molecules to afford the anhydrous [{Cu(cyclam)}_2_(*A*-H_2_As_2_Mo_6_O_26_)] (**2**). Dehydration involves an unprecedented solid-state isomerization of the POM anion, together with changes in the Cu^II^ bonding scheme that lead to a microporous supramolecular framework with gas sorption ability. These structural modifications are reflected in electron spin resonance (ESR) spectroscopic analyses.

## Experimental Section

### Materials and Methods

All reagents were purchased from commercial sources and used without further purification. Carbon, nitrogen, and hydrogen were determined on a EuroVector EA 3000 CHNSO analyzer. Fourier transform-infrared (FT-IR) spectra were recorded on KBr pellets using a Shimadzu FTIR-8400S spectrophotometer (400–4000 cm^–1^ range, 4 cm^–1^ resolution, 20 scans per spectrum). Thermogravimetric analyses (TGA) were carried out from room temperature to 600 °C at a rate of 5 °C min^–1^ on a Mettler-Toledo TGA/SDTA 851^e^ thermobalance under a 50 cm^3^ min^–1^ flow of synthetic air. Powder X-ray diffraction (PXRD) patterns were collected on a Bruker D8 Advance diffractometer operating at 40 kV/40 mA and equipped with Cu Kα radiation (λ = 1.5418 Å), a Vantec-1 PSD detector, an Anton Parr HTK2000 high-temperature furnace, and Pt sample holder. Data sets were acquired in 2θ steps of 0.033° in the 5 ≤ 2θ ≤ 40 range (a) from 30 to 600 °C every 10 °C, (b) from 30 to 150 °C every 5 °C, and (c) from 150 to 30 °C every 5 °C. Electron spin resonance (ESR) spectra were recorded on Bruker ELEXSYS 500 (superhigh-Q resonator ER-4123-SHQ) and Bruker EMX (ER-510-QT resonator) continuous wave spectrometers for the Q- and X-bands, respectively.

### Synthesis of [Cu(cyclam)(H_2_O)_2_]_0.5_[{Cu(cyclam)}_1.5_{*B*-H_2_As_2_Mo_6_O_26_(H_2_O)}]·9H_2_O (**1**), [{Cu(cyclam)}_2_(*A*-H_2_As_2_Mo_6_O_26_)] (**2**), and [{Cu(cyclam)}_2_(*A*-H_2_As_2_Mo_6_O_26_)]·6H_2_O (**2h**)

A mixture of CuCl_2_·2H_2_O (0.170 g, 1 mmol) and cyclam (0.040 g, 0.2 mmol) in water (15 mL) was added dropwise to an aqueous solution (10 mL) of (NH_4_)_6_Mo_7_O_24_·4H_2_O (0.213 g, 0.2 mmol) and KH_2_AsO_4_ (0.060 g, 0.3 mmol) previously adjusted to pH = 2.5. Then, the pH of the mixture was set to 4 with aqueous 3 M NaOH, and the cloudy solution was stirred for 1 h at 50 °C. After cooling down to room temperature, it was filtered off to remove a dark pink solid and allowed to evaporate in an open container at room temperature. Pink block-like crystals of **1** suitable for X-ray diffraction were isolated after 4 days. Yield: 40 mg (31% based on Mo). Elemental analysis, % calcd (found) for C_20_H_72_As_2_Cu_2_Mo_6_N_8_O_37_: C 12.85 (12.62), H 3.88 (3.74), N 5.99 (5.78). FT-IR (KBr, cm^–1^): 3430 (vs), 3225 (vs), 3178 (vs), 3132 (vs), 2939 (s), 2877 (s), 1627 (m), 1465 (m), 1427 (m), 1296 (w), 1242 (w), 1095 (m), 1064 (w), 1003 (w), 941(m), 899 (vs), 837 (s), 658 (s), 540 (w). The anhydrous **2** can be prepared by heating a sample of **1** at 100 °C in an oven for 1 h. However, crystals suitable for X-ray diffraction were only obtained by placing single-crystals of **1** under high vacuum (10^–5^ Pa) for 1 h. The hydrated **2h** derivative was isolated by exposing crystals of **2** to air for 1 day.

### X-ray Crystallography

Crystallographic data for compounds **1** and **2** are summarized in Table 1. Intensity data were collected on an Agilent Technologies SuperNova diffractometer equipped with an Eos CCD detector, mirror-monochromated Mo Kα radiation (λ = 0.71073 Å) and an Oxford Cryostream 700 PLUS temperature device. For the measurement of the anhydrous **2**, single crystals of **1** were kept under high vacuum for 1 h to ensure full conversion. Immediately afterward, the selected crystal was covered with Paratone oil, placed under the N_2_ stream of the diffractometer and quenched to 100 K to proceed to the full data acquisition. Data frames were processed (unit cell determination, analytical absorption correction with face indexing, intensity data integration, and correction for Lorentz and polarization effects) using the CrysAlis Pro software package.^35^ The structures were solved using OLEX2 1.3^36^ and refined by full-matrix least-squares with SHELXL-2018/3.^37^ Final geometrical calculations were carried out with PLATON^38^ as integrated in WinGX.^39^ Bond valence sum (BVS) calculations^40^ were carried out using the BVSumCalc program. Thermal vibrations were treated anisotropically for all the non-H atoms. In the case of **2**, thermal ellipsoids belonging to some C, N, and O atoms were restrained using ISOR commands. Hydrogen atoms of the organic ligands were placed in calculated positions and refined using a riding model with standard SHELXL parameters, whereas those belonging to protonation sites within the inorganic skeleton of the POM anion were located in the Fourier map and O–H bond lengths were restrained to 0.84(2) Å. Up to 13 positions suitable for water molecules of hydration were located in the Fourier map of **1**, and their occupancies were initially refined without restrictions. The resulting total number of 9.5 molecules per POM anion was fixed to 9 during the final refinement, in good agreement with TGA experiments. Crystals of **2h** were of inferior quality compared to those of **1** and **2**, and thus, they only allowed for a preliminary structural resolution.

**Table 1:** Crystallographic Data for **1** and **2**

	**1**	**2**
formula	C_20_H_72_As_2_Cu_2_Mo_6_N_8_O_37_	C_20_H_50_As_2_Cu_2_Mo_6_N_8_O_26_
fw(g mol^–1^)	1869.41	1671.24
crystal system	triclinic	triclinic
space group	*P*-1	*P*-1
*a* (Å)	13.3715(5)	8.5999(18)
*b* (Å)	13.8812(6)	12.110(3)
*c* (Å)	17.5569(7)	12.2460(16)
α (deg)	69.963(4)	95.835(15)
β (deg)	78.525(3)	99.709(15)
γ (deg)	67.337(4)	98.024(19)
*V*(Å^3^)	2816.7(2)	1234.5(4)
ρ_*c*alcd_(g cm^–3^)	2.204	2.248
μ (mm^–1^)	3.297	3.731
reflections		
collected	18787	8259
unique (*R*_int_)	10126 (0.030)	4493 (0.095)
obs. [*I*> 2σ(*I*)]	8477	2354
parameters	665	682
restraints	4	24
*R*(*F*)^a^ [*I*> 2σ(*I*)]	0.045	0.115
*wR*(*F*^2^)^b^ [all]	0.116	0.341
GoF	1.053	1.045

a *R*(*F*) = Σ||*F*_o_ – *F*_c_||/Σ|*F*_o_|.

b _*w*_*R*(*F*^2^) = {Σ[*w*(*F*_o_^2^ – *F*_c_^2^)^2^]/Σ[*w*(*F*_o_^2^)^2^]}^1/2^.

### Gas Sorption Measurements and Simulation Details

The porous texture of **2** was characterized through the physical adsorption of N_2_ and CO_2_ gases. Gas sorption isotherms were collected using a Quantachrome Autosorb-iQ-MP device. Samples were degassed at 70 °C under high vacuum for 24 h prior to gas adsorption measurements. Nitrogen isotherms were acquired at 77 K while carbon dioxide physisorption data was recorded at 273 K. Force-field based grand canonical Monte Carlo (GCMC) simulations of single-component (N_2_ and CO_2_) adsorption were carried out using the SORPTION module included in the Accelrys “Materials Studio” package.^41^ The theoretical background of GCMC simulations is described in detail elsewhere.^42^ Simulations were carried out under the same conditions as those selected for experimental gas sorption measurements (*P* < 1 bar; N_2_ at 77 K, CO_2_ at 273 K) and involved 4 million equilibration steps and 6 million production steps. The pore size distribution was computed using a Monte Carlo procedure implemented in a previously reported code,^43^ in which universal force field Lennard–Jones (LJ) parameters are used to describe adsorbent atoms while a probe of incremental size explores the free volume. In all simulations, dispersive and electrostatic interactions were taken into account. For more details see the Supporting Information.

## Results and Discussion

### Synthetic Aspects

Compound **1** was synthesized by mixing an aqueous solution of CuCl_2_ and the cyclam ligand with a commercial heptamolybdate salt and the heteroatomic source KH_2_AsO_4_ at pH = 4. Single-crystals were obtained in a moderate yield (∼30%) by slow evaporation of the final solution at room temperature for a few days. FT-IR spectroscopy allowed a preliminary identification of the solid as a {Cu(cyclam)}^2+^/molybdoarsenate hybrid (Figure S1) by the collection of bands ascribed to the {Cu(cyclam)}^2+^ complex^44^ in the metal–organic region of the spectrum above 1100 cm^–1^ and characteristic signals of the POM anion below this wavenumber. Vibrational bands centered at 941, 899, 837, and 658 cm^–1^ could be originating from ν(Mo=O_t_), ν(As–O), and ν(Mo–O_b_–M) (M = Mo, As) stretching modes, respectively, which suggests the presence of the [H_*x*_As_2_Mo_6_O_26_]^(6–*x*)^-cluster.^32^ Although they do not show the same molecular formula, up to three different structural so-called isomers have been reported to date for this anion, namely, [*A*-H_2_As_2_Mo_6_O_26_]^4–^ and *B*- and [*B*′-H_2_As_2_Mo_6_O_26_(H_2_O)]^4–^. The *A*-isomer has been long known and both its combination with transition metal complexes as well as the organic derivatization through organoarsonate groups has extensively been exploited.^34,45^ In contrast, there are only a few reports related to *B*/*B*′-type isomers (see their molecular structures in Figure S2).^33^ Unfortunately, the IR spectra belonging to different isomers are virtually identical,^33^ and hence, it prevented us from its preliminary spectroscopic identification.

Reaction temperature affects the amount and crystallinity of the final product. When no additional heating was applied and reaction took place at room temperature, a lower reaction yield was observed whereas moderate heating resulted in solid samples with lower crystallinity. Thus, slight warming at 50 °C was selected as the best choice. On the other hand, neither the cation of arsenate salts (sodium, potassium, ammonium) nor the anion of the copper(II) ion (chloride, nitrate, sulfate, acetate) used as starting reactants has any remarkable effect in the synthetic process.

### Thermostructural Behavior

Thermostructural behavior in **1** was studied by means of thermogravimetric (TGA) and variable-temperature powder X-ray diffraction (VT-PXRD) analyses. First, the TGA curve (Figure S3) revealed a dehydration process below 100 °C (found, % 10.9; calcd for 11 H_2_O, % 10.6), which is followed by a plateau that could be associated with the presence of a stable anhydrous phase that extends from 100 to 225 °C. Thermal decomposition of the anhydrous form should proceed through the combustion of two organic cyclam ligands and the breakdown of the POM framework to lead to the final residue at ∼460 °C. PXRD analyses (Figure S4) allowed us to identify the final residue as a mixture of 11 MoO_3_ (PDF 01-076-1003)/CuMoO_4_ (PDF 01-085-1530)/Cu_3_(AsO_4_)_2_ (PDF 01-078-1866). The calculated mass for this residue is in good agreement with the observed values (found, % 61.2; calcd, % 61.3) and implies the loss of 1/2 As_2_O_5_ molecule in the thermal decomposition process.

**Figure 1:**
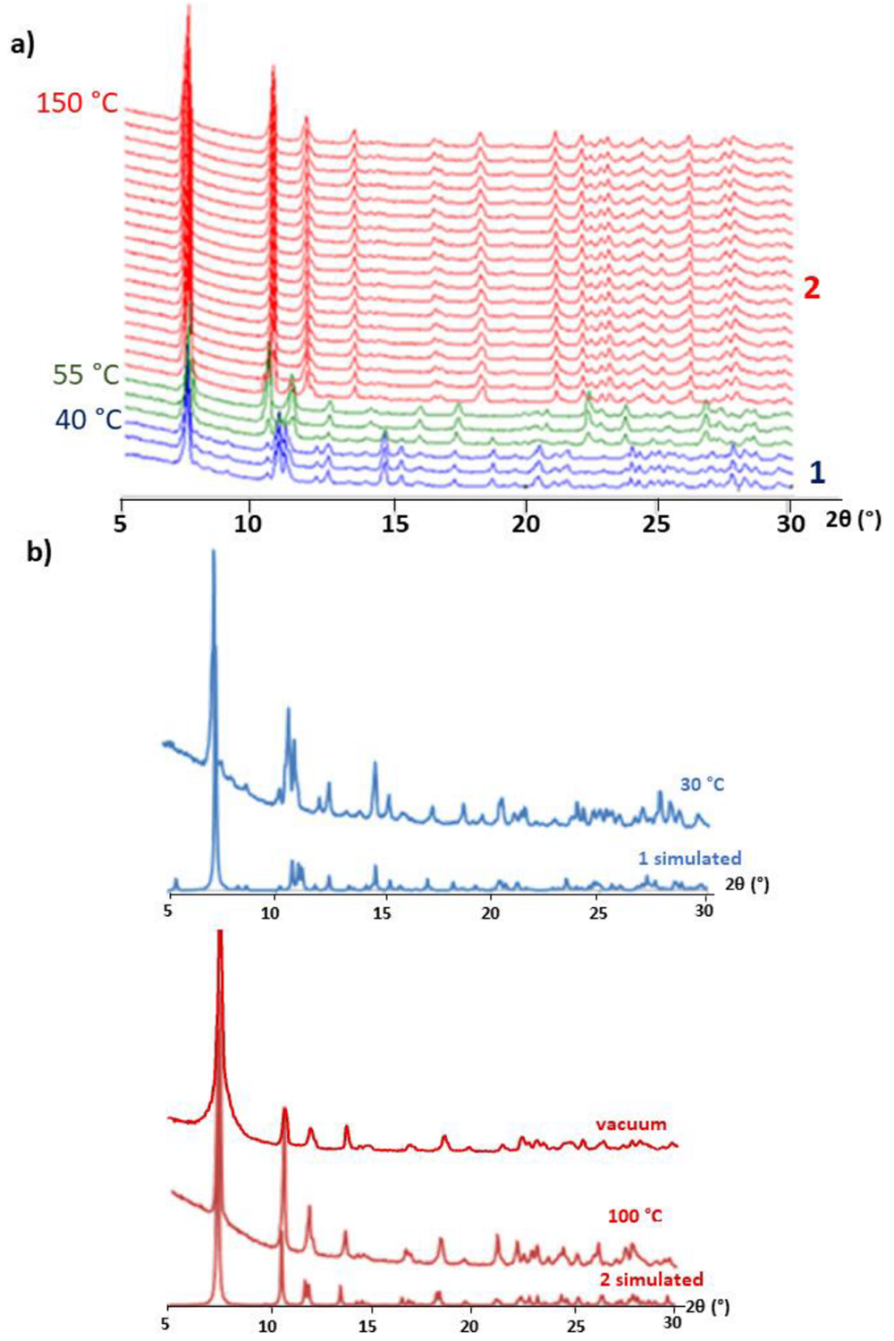
(a) Variable-temperature PXRD patterns of **1** when heating from room temperature to 150 °C. (b) Comparison of the patterns collected at 30 °C, 100 °C, and under high vacuum (10^–5^ Pa) with those simulated from scXRD data of **1** and **2**, respectively.

In order to determine whether **1** is able to exhibit a comparable thermostructural behavior to that displayed by related {Cu(cyclam)}^2+^/POM systems in which the crystallinity is retained all along the dehydration process,^12,13,21,22^ VT-PXRD analyses were performed from room temperature to 600 °C (Figure S5). These studies showed that our hybrid compound is able to maintain its crystallinity up to 250 °C, which is in good agreement with the thermal stability range observed in TGA measurements. The patterns above 450 °C are virtually identical to that acquired for the final residue of the thermal analyses. Close inspection of the patterns recorded every 5 °C up to 150 °C (Figure 1) revealed that two different structural transitions are clearly observed upon heating. First, **1** rapidly transforms into a partially dehydrated phase at 40 °C as indicated by relevant modifications in the positions of the most intense diffraction maxima located at low 2θ angles. In particular, the most intense maxima at 7.4° is retained, whereas those at 10.7 and 11.0° in **1**, shift toward 10.4 and 11.2° 2θ values. Similarly, signals at 14.7 and 15.3° are completely vanished and an additional group of three distinctive low intensity maxima centered at ∼23° appear. Above 60 °C, a third crystalline phase is fully formed which continues displaying the most intense signal at 7.4° but in contrast shows three characteristic maxima at 10.6, 11.8, and 13.6° and a subsequent series of low intensity signals at higher 2θ angles (21–30° range). A virtually identical pattern is maintained upon total dehydration above 100 °C, which implies the presence of a stable and crystalline anhydrous form (noted as **2**).

### Crystal Structure of **1**

Single crystal X-ray diffraction (scXRD) analyses revealed that compound **1** crystallizes in the triclinic space group *P*-1, and its asymmetric unit contains one [*B*-H_2_As_2_Mo_6_O_26_(H_2_O)]^4–^ anion (*B*-As_2_Mo_6_), one {Cu(cyclam)}^2+^ complex (Cu1A) in a general position, two halves of centrosymmetric {Cu(cyclam)}^2+^ (Cu1B) and {Cu(cyclam)(H_2_O)_2_}^2+^ (Cu1C) complexes as well as 9 water molecules of hydration disordered over 13 crystallographic sites (Figure S6). The *B*-As_2_Mo_6_ anion can be best described as a bent, six-membered ring constituted by four edge-sharing {MoO_6_} octahedra which share corners with a {Mo_2_O_8_(H_2_O)} fragment. Bond valence sum (BVS) calculations confirmed that the three bridging ligands that allow the face sharing in the latter fragment are two oxo groups and one water molecule (BVS = 0.23 for the water molecule, whereas values above 1.85 were found for the oxo groups). In addition, the anion is capped on opposite faces by two protonated arsenate groups in such a way that the coordination water molecule establishes a strong intramolecular hydrogen bond (O_w_···O_As_ = 2.689(7) Å) with one of those {AsO_4_H} moieties.

**Figure 2:**
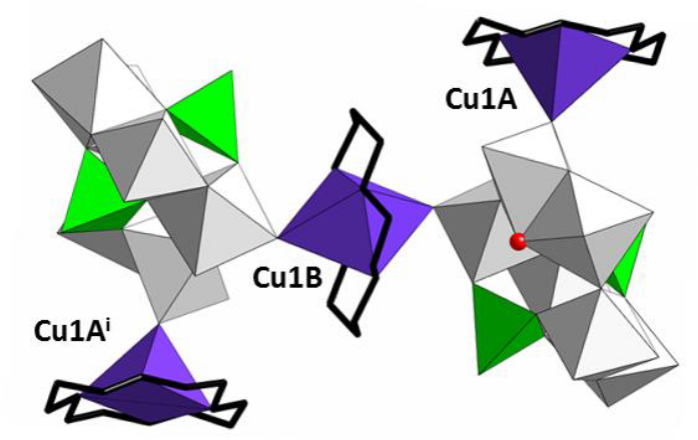
Covalent [μ-Cu(cyclam){(Cu(cyclam)(*B*-As_2_Mo_6_O_26_(H_2_O)}_2_]^2–^ building blocks in **1**. Color code: MoO_6_, gray octahedra; AsO_4_H, green tetrahedra; CuN_4_O_2_ and CuN_4_O, purple polyhedra; C, black stick; water molecules, red spheres. Symmetry code: (i) −*x*, 1 – *y*, 1 – *z*.

Covalent and centrosymmetric [μ-Cu(cyclam)-{(Cu(cyclam)(*B*-As_2_Mo_6_)}_2_]^2–^ building blocks can be found in the crystal structure of **1** (Figure 2), in which the two POM units display one Cu1A antenna complex each grafted to their surface and an additional Cu1B fragment that bridges contiguous anions through axial coordination to terminal O_POM_ atoms. In contrast, the Cu1C cation acts as a charge compensating unit. Thus, the Cu^II^ center in Cu1A exhibits a square pyramidal geometry with the four N atoms of the cyclam ligand in equatorial positions and one apical O_POM_ atom (Cu1A–O6B, 2.321(5) Å; Cu1A···O3A^i^, 3.091(7) Å; symmetry code, i: 1 + *x*, *y*, *z*) and the remaining two complexes are in an elongated octahedral coordination environment, where either O_POM_ atoms (Cu1B) or aqua ligands (Cu1C) occupy axial positions. All the bond lengths are in the expected range (Table S1), and cyclam ligands show the most stable trans-III configuration^44^ with N–H bonds from propylene bridged pairs of N atoms pointing to opposite directions (Figure S7).

**Figure 3:**
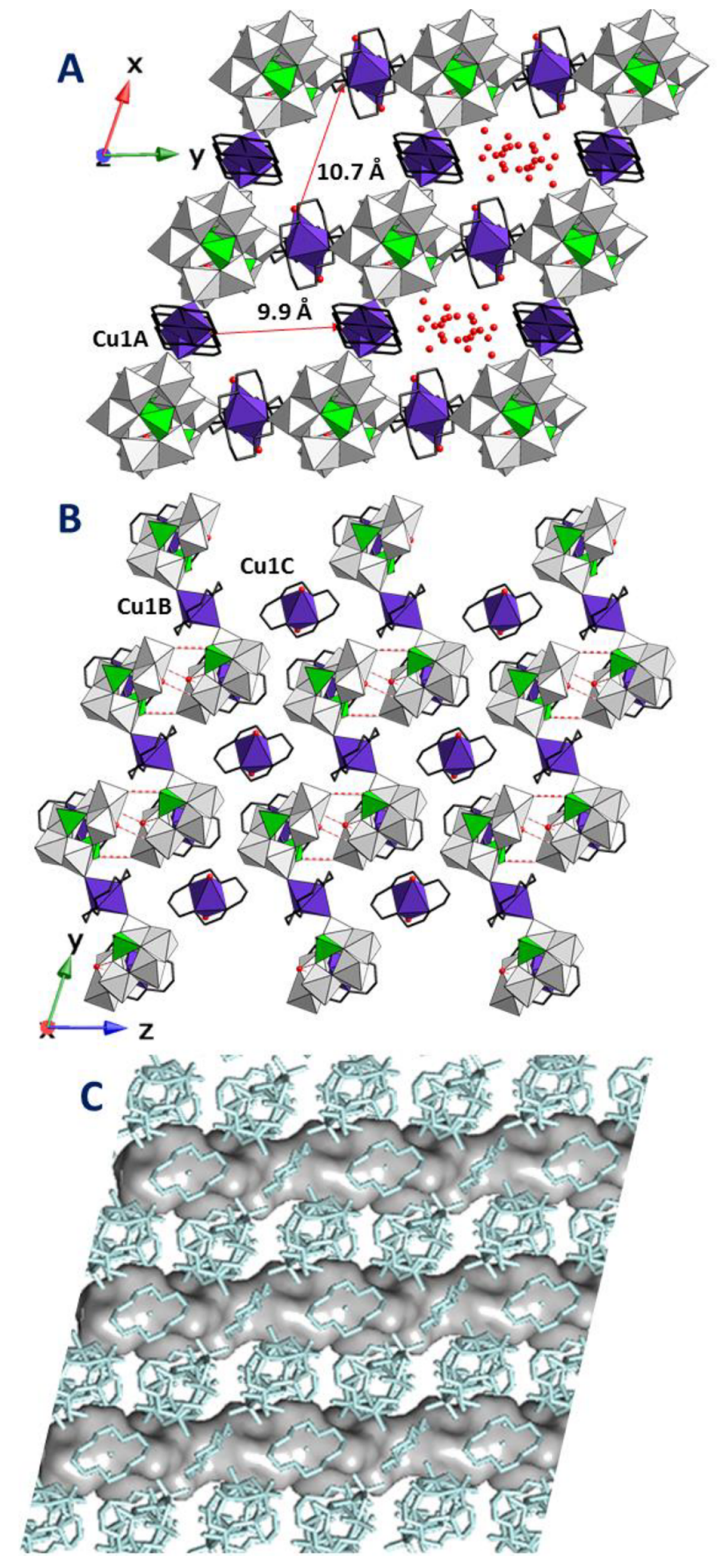
(a) View of the crystal packing of **1** along the crystallographic *z* axis, together with the shortest N···N distances within the channels. (b) Projection of the crystallographic *yz* plane representing hydrogen-bond interactions as dashed red lines. Hydration water molecules are omitted for clarity. (c) Surface representation of the solvent accessible channels running along the crystallographic *z* axis.

Dimeric POM/metal–organic building-blocks are connected by two pairs of hydrogen-bonds established between *B*-As_2_Mo_6_ units which involve one {AsO_4_H} group together with the coordination water molecule and terminal O atoms. This results in chainlike arrangements running parallel to the crystallographic *y* axis, which interact with each other through the Cu1C complex via O_w_–H···O_POM_ and C–H···O_POM_ type contacts (Figure S8) and lead to supramolecular hybrid layers in the crystallographic *yz* plane. The Cu1A antenna complexes point toward above and below these layers, facilitating their stacking via hydrogen bonding (Table S2), which generates a three-dimensional supramolecular open framework with squarelike channels parallel to the *z* axis and an approximate cross-section of 9.9 × 10.7 Å^2^ (N1A···N1A × N1C···N1C). All the hydration water molecules are hosted in these solvent accessible voids that account for a total volume of 585.9 Å^3^ and correspond to ∼21% of the unit cell volume, as calculated by PLATON (Figure 3).

### SCSC Transformation from **1** to **2**

Single crystals of **1** were slowly heated in an oven at a rate of 1 °C min^–1^ up to 50, 70, and 100 °C and subsequently quenched to 100 K to determine whether high temperature phases found in variable temperature PXRD studies can be analyzed by this technique. In contrast to our latest reports on POM/{Cu(cyclam)} systems,^21,22^ the single crystallinity is not preserved upon evacuation of water molecules, and hence, it prevented us from performing scXRD experiments on thermally activated samples. We tried to overcome these difficulties by dehydrating crystals of **1** under vacuum (*P* = 10^–5^ Pa) at room temperature for 1 h, and luckily, we were able to carry out a full data acquisition for the anhydrous **2** at 100 K. In fact, the PXRD pattern of **1** exposed to high vacuum is virtually identical to both the PXRD pattern recorded at 100 °C and the simulated one from the single crystal data for **2** (Figure 1b). However, the release of water molecules cannot be controlled when using high vacuum, and thus, it precluded us from being able to study the intermediate phase observed in the 40–55 °C temperature range.

**Figure 4:**
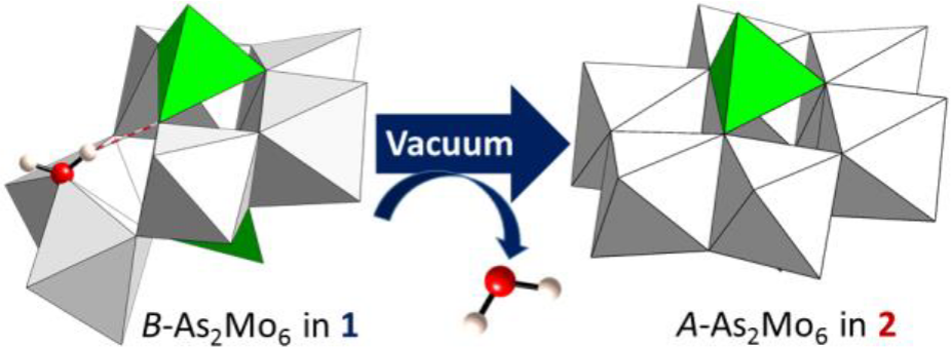
Detail of the SCSC cluster transformation upon dehydration from {*B*-H_2_As_2_Mo_6_O_26_(H_2_O)} in **1** to {*A*-H_2_As_2_Mo_6_O_26_} in **2**. Intramolecular hydrogen bond, dashed red line.

The anhydrous **2** also crystallizes in the *P*-1 triclinic space group with similar unit cell parameters except for the *a* unit cell parameter (*c* in **1**) and total cell volume, which are reduced to one-half of their value in **1** due to an overall increase of the symmetry. Thus, the new asymmetric unit only contains one-half of the [*A*-H_2_As_2_Mo_6_O_26_] (*A*-As_2_Mo_6_) anion and two halves of {Cu(cyclam)}^2+^ complexes. Total dehydration results in major modifications within the inorganic polyoxoanion. The [*B*-H_2_As_2_Mo_6_O_26_(H_2_O)] anion transforms into the [*A*-H_2_As_2_Mo_6_O_26_] isomer upon removal of the coordination water molecule and subsequent condensation of the {Mo_2_O_8_} unit to adjacent addenda metal centers via edge-sharing (Figure 4). This transition involves an important change in the relative arrangement of Mo centers from a bent configuration in the *A* isomer to a planar form similar to that displayed by the well-known [α-Mo_8_O_26_]^4–^ in *B*, in such a way that it gets constituted by a ring of six edge-sharing {MoO_6_} units, capped by one protonated arsenate group from each side in an ideal *D*_3*d*_ symmetry. The solution interconversion between both isomeric forms was previously observed by Pope and co-workers,^45^ but the solid-state transition reported herein is unprecedented and represents one of the scarce examples of SCSC transformations in POM-based systems that imply the rearrangement of the cluster skeleton.

**Figure 5:**
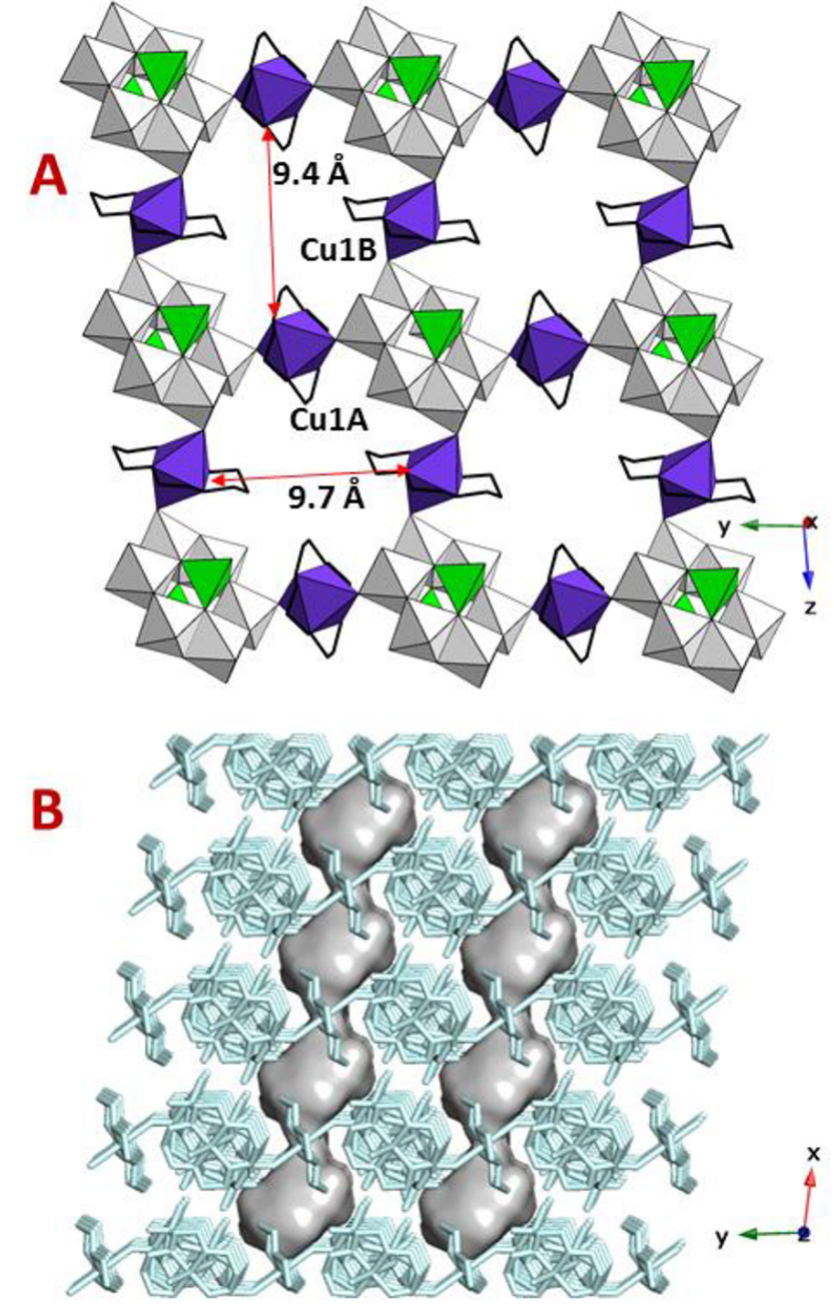
(a) Projection of the hybrid layer on the *yz* plane for **2** together with the shortest N···N distances within the channels. (b) Crystal packing of **2** viewed along the crystallographic *z* axis and surface representation of structural voids running along the crystallographic *x* axis.

This transition is also associated with modifications in the Cu(II) coordination spheres in such a way that only two crystallographically independent octahedral centers can be found in **2**. Each *A*-As_2_Mo_6_ unit is linked to four neighboring clusters through bridging {Cu(cyclam)}^2+^ moieties in the crystallographic *yz* plane, which results in covalent cluster/metalorganic layers with squarelike grids by retaining the microporous nature of the parent phase. The stacking of these hybrid layers generates empty channels running parallel to the crystallographic *x* axis constituted by large voids with an approximate cross-section of 9.4 × 9.7 Å^2^ connected by narrow necks (Figure 5). The overall solvent accessible void accounts for a total volume of 161.8 Å^3^ and corresponds to ∼13.1% of the unit cell volume, as calculated by PLATON.

### Reversibility of the SCSC Transformation

**Figure 6:**
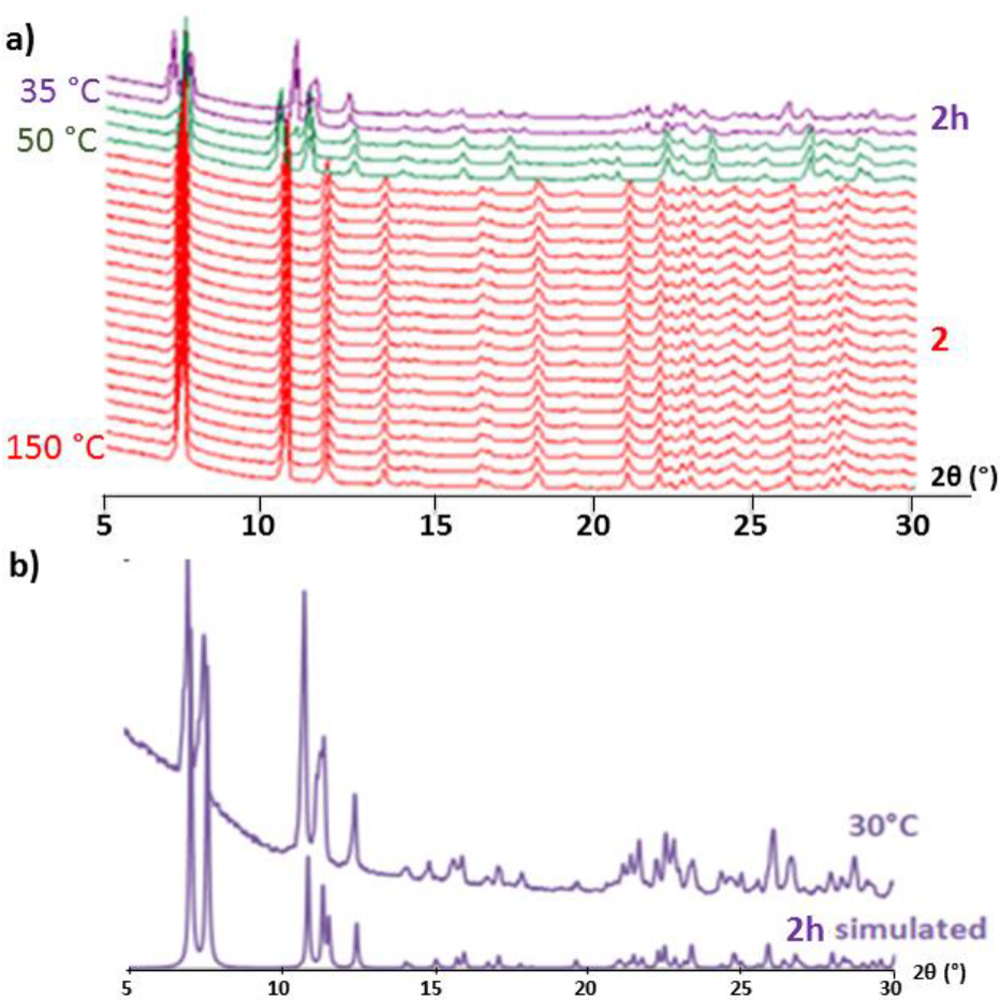
(a) Variable-temperature PXRD patterns of **2** when cooling from 150 °C to room temperature. (b) Comparison of the pattern collected at 30 °C with that simulated from scXRD data of **2h**.

A combination of thermal and diffractometric analyses allowed us to determine the reversibility of the SCSC transformation. First, a powdered sample of **1** was heated at 150 °C for 1 h, and then, PXRD patterns were acquired every 5 °C upon cooling down to room temperature (Figure 6a). As expected, the pattern recorded at 150 °C compares well with that simulated from scXRD data of the anhydrous **2** and no significant variation is observed neither in the positions nor in the intensities of the most intense diffraction maxima upon cooling down to 55 °C. Below this temperature, the formation of two additional crystalline phases can be noticed. Close inspection revealed one stable phase from 50 to 40 °C characterized by the most intense maxima at ∼2θ 7.4°, 10.4°, and 11.3°, which resembles the intermediate phase found in the VT-PXRD experiments carried out for **1** (see the Thermostructural Behavior section). These patterns clearly differ from those generated below this temperature (**2h**) with additional signals at ∼7.1 and 10.7°. Both are different from that of the previously synthesized compound **1**, which demonstrates the irreversible nature of the phase transition.

To determine whether this transformation is promoted by the hydration of the anhydrous phase, thermogravimetric analyses were performed on a sample of **2**, which was previously kept at room temperature in an open atmosphere for 24 h/72 h to ensure full rehydration. Both TGA curves revealed a virtually identical dehydration stage that proceeds from room temperature up to ∼110 °C and involve the release of 6 H_2_O molecules (calcd 6.5%, found 6.2%), followed by a thermal stability range that extends up to ∼265 °C (Figure S9). In addition, the reversibility of such transformation was assessed by combined TGA and PXRD analyses on crystalline samples of **2h** previously dehydrated at 100 °C for 1 h in an oven and kept to hydrate in an open container for 1 day. All these studies indicate that (i) **2** adsorbs ambient moisture upon air exposure to transform into **2h**; (ii) complete hydration takes place in 24 h; and (iii) this transition is fully reversible.

Encouraged by the interesting crystal dynamics displayed by **1**, we tried to carry out scXRD experiments on the hydrated **2h**. Different crystal batches of **1** were thermally dehydrated and left to adsorb ambient moisture for 24 h, but unfortunately the poor quality of the crystals tested prevented us again from collecting appropriate data for the elucidation of the crystal structure. Similar results were obtained when crystals were dehydrated under vacuum. However, one of those attempts carried out under this experimental setup allowed us to perform a full data acquisition at 100 K on a phase for which the simulated PXRD pattern compares well with that recorded for **2h** (Figure 6b). The structural resolution was of inferior quality (see the Supporting Information for the CIF file with the preliminary structural resolution),^46^ and hence, we decided not to deposit the crystallographic data in the Cambridge Structural Database (CSD).^47^ In spite of all these drawbacks, both the POM anion and cationic metal–organic complexes were conveniently located in the Fourier map, but only two hydration water molecules (disordered over three crystallographic positions) were found, far from the total amount of six determined in TGA analyses. The preliminary structural resolution, together with thermal analyses, strongly suggests the general formula [{Cu(cyclam)}_2_(*A*-H_2_As_2_Mo_6_O_26_)]·6H_2_O for the fully hydrated **2h** which displays an open hybrid framework, similar to that shown by **2** but with the guest hydration water molecules filling the structural voids. The most remarkable difference is the lengthening of the Cu1B···Cu1B intralamellar distance and the related unit cell parameter *c* by ∼0.7 Å, which originates from the rotation of the POM anion on ∼8° with respect to the crystallographic *xy* plane (Figure S10). Hydration also results in a swelling of the structure with a slight increase of the total solvent accessible void in comparison to **2** up to 188.4 Å^3^ (14.7% of the total cell volume), as calculated by PLATON.

### EPR Spectroscopy

Phase transformation from **1** to **2** can be easily followed by electron paramagnetic resonance (EPR) spectroscopy, because the structural transition involves changes in the EPR signals. Room temperature X-band (9.40 GHz) spectrum of **1** shows a quasi-isotropic signal centered at ∼3220 G together with two small shoulders at ∼2780 and 3420 G. The shape of this signal remains virtually unaltered upon lowering the temperature down to 5 K, in good agreement with that expected for a paramagnetic system (Figure S11). In contrast, the signal recorded at Q-band (33.92 GHz) is much more complex, and besides the central line, two well-defined shoulders separated by 200 G are clearly observed at the low-field region of the spectrum. All these small signals can be ascribed to the parallel component of the hyperfine structure originating from the coupling of electronic (*S* = 1/2) and nuclear spins (*I* = 3/2) of magnetically isolated Cu^II^ ions. The intense central line is apparently isotropic at X-band but it shows less symmetry at Q-band. Thus, contributions from both isolated and magnetically coupled Cu^II^ ions with different environments and/or orientations can be deduced from the latter spectrum.

The X-band spectrum can be reasonably good fitted if both signals are considered (Table 2 and Figure S12) and the signal corresponding to magnetically isolated Cu^II^ ions (Signal 1) is in good agreement with the hyperfine structure observed at Q-band. However, we were not able to find a *g* tensor that allows the simultaneous fitting of the central line (Signal 2) of both spectra, which suggests that it corresponds to the average of contributions from different Cu^II^ centers. The *g* and *A*_∥∥_ values determined from Signal 1 are characteristics for Cu^II^ ions in an axially elongated environment displaying a N4-equatorial coordination, whereas the average *g* value for Signal 2 indicates that magnetic orbitals of the exchange coupled metal centers are mainly of the d_*x*^2^__*–y*^2^_ nature.

**Table 2:** Spin Hamiltonian Parameters *g* and *A* for Compound **1**

	X-band	Q-band
Signal 1		
*g*_∥_	2.180	2.180
*g*_⊥_	2.057	2.057
*A*_∥_ (× 10^–4^ cm^–1^)^a^	202	210
*A*_⊥_ (× 10^–4^ cm^–1^)	<20	<20
Signal 2		
⟨*g*⟩	2.085	

a Small differences arising from the better resolution of Q-band.

**Figure 7:**
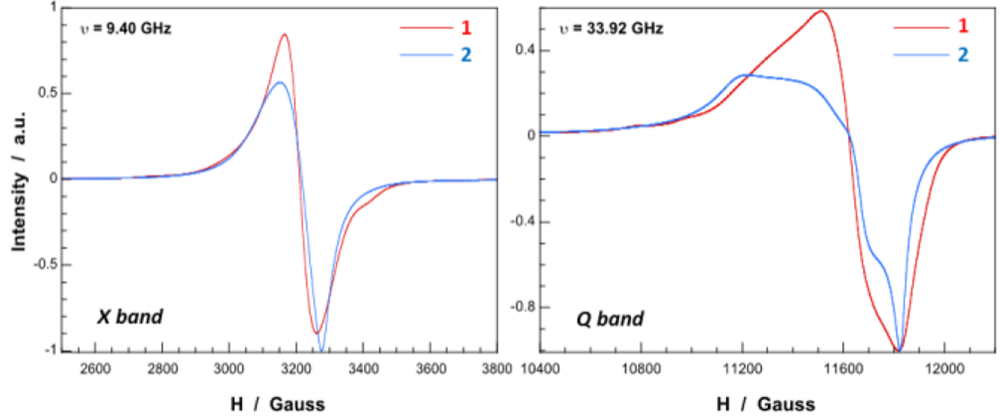
X- and Q-band EPR spectra of **1** and **2**.

For comparison, spectra registered for **2** are very complex and exhibit signals from different magnetically coupled species. It is worth noting that dehydration from **1** to **2** promotes the magnetic coupling of previously isolated copper centers in such a way that the hyperfine structure observed in **1** is completely vanished (Figure 7).

### Gas Sorption Properties

**Figure 8:**
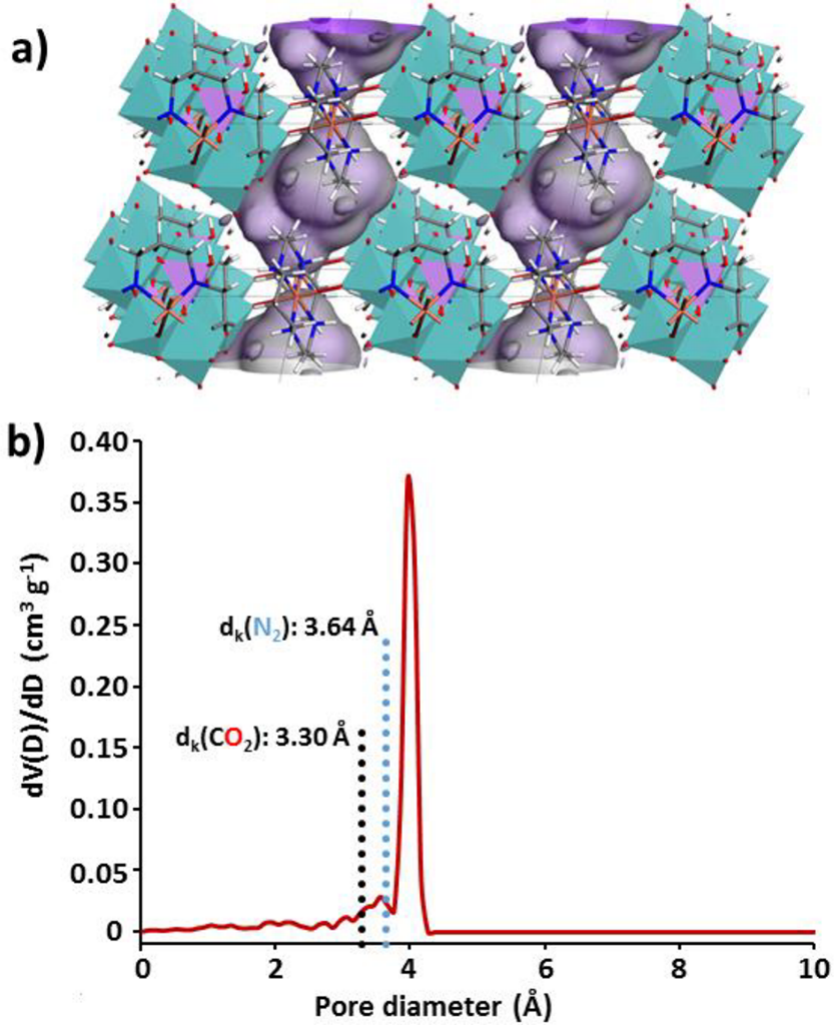
(a) Connolly surface of **2** depicting the accessible surface created for a N_2_ sized molecular probe and (b) computed pore size distribution of **2** (kinetic diameters for CO_2_ and N_2_ are indicated with dotted black and blue lines, respectively).

Single-crystal XRD studies proved that the supramolecular open-framework in **1** can retain its porosity throughout the structural transformation that the system undergoes upon evacuation of guest solvent molecules. The anhydrous phase **2** displays empty channels with cross sections larger than the size of N_2_ and CO_2_ molecules and hence, potentially accessible for such kind of small gaseous species. The calculated accessible surface plot created for a N_2_ sized molecular probe (Figure 8a) shows that the pore system of **2** consists of a distribution of isolated wide cavities running along the [100] direction and connected by narrow necks, which seems to be close to percolation. In fact, the calculated pore size distribution (Figure 8b) shows that major cavities (∼4.1 Å) are wide enough to host common adsorbate molecules such as H_2_, N_2_, CO, and CO_2_. However, necks seems to be narrow enough (∼3.6 Å) to preclude the diffusion of species that exhibit greater kinetic radii (N_2_, 3.64 Å; CO, 3.76 Å), whereas they should allow the sorption of smaller molecules (CO_2_, 3.30 Å; H_2_, 2.89 Å). To elucidate whether these observations can be experimentally reproduced, we carried out N_2_ and CO_2_ sorption experiments on a crystalline sample of **1** activated under vacuum at 70 °C for 24 h to ensure the evacuation of all the guest water molecules. The resulting solid was confirmed to be the anhydrous phase **2** on the basis of PXRD analyses (Figure S15).

**Figure 9:**
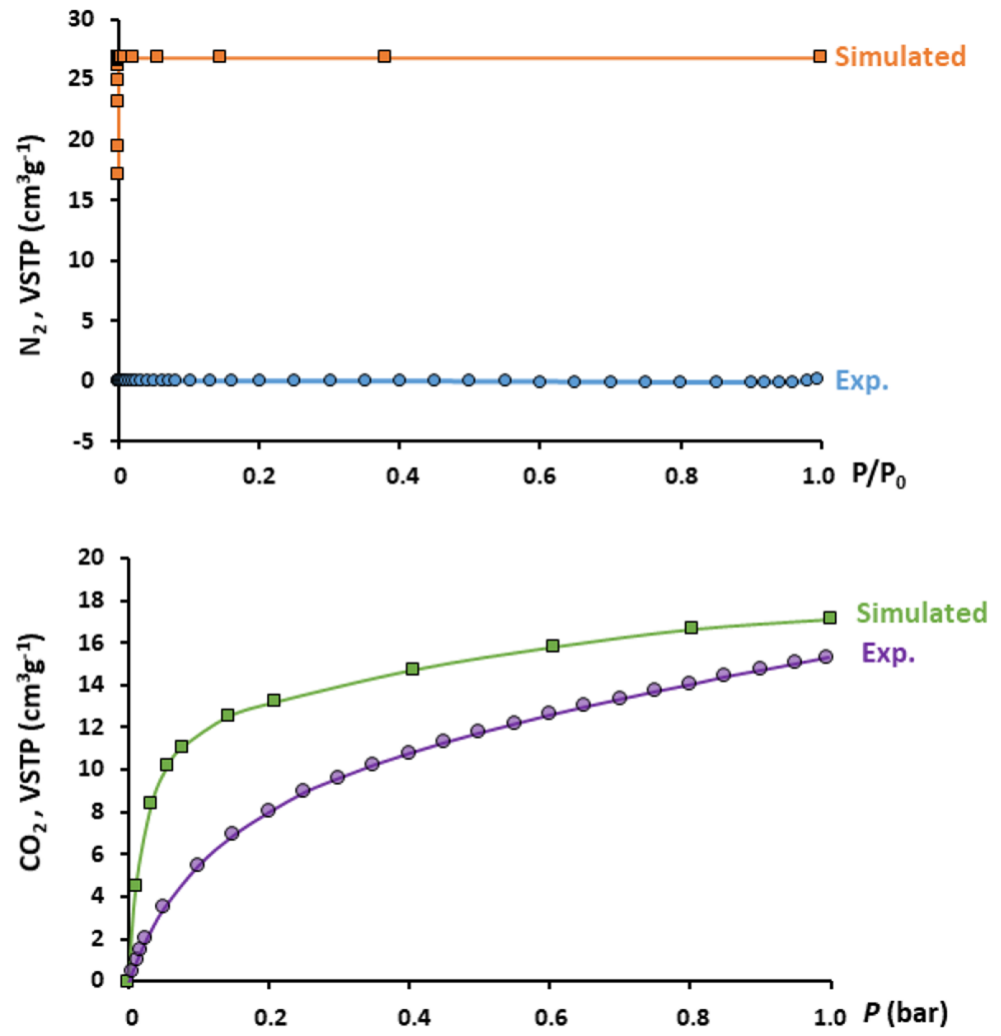
Experimental and simulated adsorption isotherms for **2**. Top, N_2_ at 77 K; and bottom, CO_2_ at 273 K.

Our experiments revealed that the experimental isotherm for **2** exhibits a negligible N_2_ adsorption, far from the simulated values that shows a potential total gas uptake equivalent to two adsorbed N_2_ molecules per molybdoarsenate(V) cluster (Figure 9a and Figure S16). However, a moderate amount of CO_2_ sorption can be experimentally observed in Figure 9b. As expected from the pore size distribution plot, compound **2** behaves as a molecular sieve by selectively adsorbing CO_2_ and excluding N_2_, which makes this material of potential interest in selective gas capture and purification technologies.

The experimental isotherm for the CO_2_ sorption process at 273 K is characteristic of a microporous material with the total uptake value (STP, 15.3 cm^3^ g^–1^) close to the simulated one (17.2 cm^3^ g^–1^). Note that the simulation somewhat exceeds the experimental uptake, especially at low pressure. Such a trend can be attributed to an overestimation of the computed charge distribution for the structural model as it has been previously reported for this kind of simulations.^48^ Gas uptakes lower than expected are usually related with samples of low crystallinity, the presence of impurities, or incomplete outgassing. However, these causes do not explain our observations because the samples used in the gas sorption measurements consist of single crystals with several hundred micron sizes, and the TGA analyses confirm an efficient outgassing. Thereby, the diffusion of CO_2_ molecules through narrow pore necks seems to be the fact that hampers the access of the gas probe to the isolated cavity system. In any case, the adsorbed amount of 1.2 molecules per molybdoarsenate cluster at *P* = 1 bar is close enough to the theoretically expected value of 1.3.

**Figure 10:**
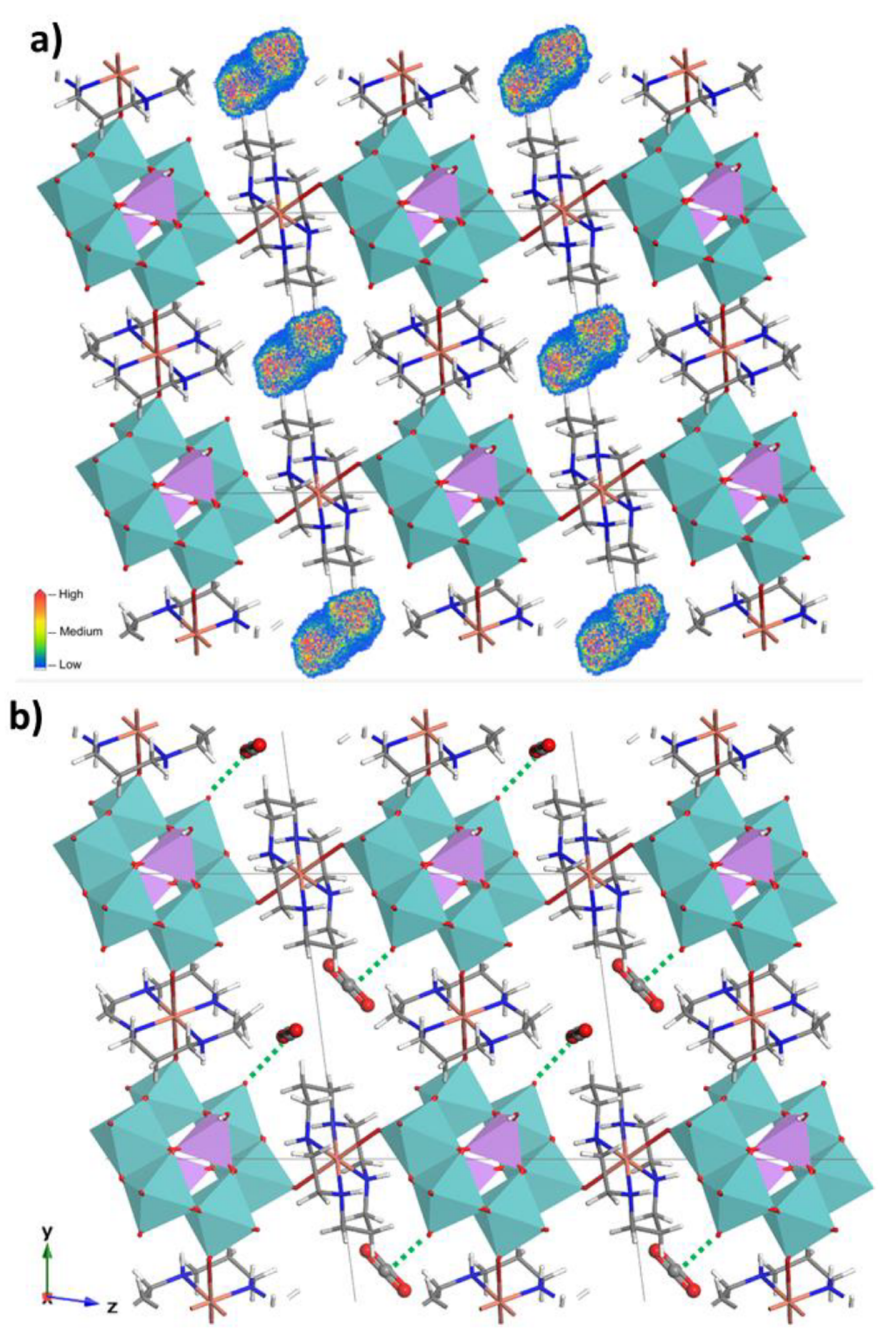
(a) Simulated average occupation profiles for CO_2_ in **2** at 1 bar and (b) selected lowest energy configuration. C_CO2_···O_POM_ interactions are depicted as dashed green lines.

To get a better understanding of the adsorption behavior of **2**, the CO_2_ average occupation profiles have been plotted at high uptakes (1 bar) as shown in Figure 10a. In the absence of any highly interacting site, such as coordinatively unsaturated positions, the adsorption sites are ascribed to the surrounding pore walls of the wide cavities. The profile displays as much as two preferential occupation sites for CO_2_ molecules within these voids that encouraged us to calculate their lowest energy configuration in **2** at saturation conditions (Figure 10b). Both CO_2_ molecules interact through their carbon atom (provided with positive charge density) with the O2A terminal oxygen atom of the molybdoarsenate(V) unit, implying relatively short C_CO2_···O_POM_ distances (2.728 and 2.894 Å, respectively).

## Conclusions

Compound [Cu(cyclam)(H_2_O)_2_]_0.5_[{Cu(cyclam)}_1.5_{*B*-H_2_As_2_Mo_6_O_26_(H_2_O)}]·9H_2_O (**1**) represents one of the scarce examples of POM-based systems that simultaneously display crystal dynamics induced by the evacuation of solvent molecules and exhibits permanent microporosity, which renders the anhydrous phase with selective gas sorption properties. The supramolecular open framework in **1** is made of discrete [μ-Cu(cyclam){(Cu(cyclam)(*B*-H_2_As_2_Mo_6_O_26_(H_2_O))}_2_]^2–^ covalent units and additional {Cu(cyclam)}^2+^ cations. Although **1** can undergo up to two thermally triggered phase transitions, it does not preserve its single-crystalline nature upon heating. In contrast, when solvent molecules are released under vacuum, the anhydrous phase [{Cu(cyclam)}_2_(*A*-H_2_As_2_Mo_6_O_26_)] (**2**) is isolated through single-crystal-to-single-crystal (SCSC) transformations. This fact reveals the key importance of the dehydration mechanism. Phase transition leads to important modifications within the inorganic cluster skeleton in such a way that an unprecedented solid-state *B* to *A* isomerization of the polyoxoanion is observed. Changes in the Cu^II^ bonding scheme upon structural transformations increases the dimensionality of the parent lattice and leads to covalent cluster/metalorganic layers by retaining the open-framework nature of **1**. Compound **2** adsorbs ambient moisture upon air exposure, and it undergoes a SCSC transformation to lead to the hydrated [{Cu(cyclam)}_2_(*A*-H_2_As_2_Mo_6_O_26_)]·6H_2_O (**2h**). The permanent porosity of **2** is confirmed by gas sorption experiments. Theoretical calculations showed that narrow necks connect wider cavities, and this hinders the adsorption of gaseous molecules greater than ∼3.6 Å, which endows the material with the capability to selectively adsorb CO_2_ over N_2_. Besides its potential as a molecular sieve for the purification of gas mixtures, the solid state cluster transformation reported in this work suggests that the isolation of novel POM anions might be possible.

## Supporting Information Available

Structural figures and tables, FT-IR spectra, PXRD patterns, TGA analyses, gas sorption experiments, and computational details (PDF)
Crystallographic information file for **2h** (CIF)

## Supplementary Material

ic1c02276_si_001.pdf

ic1c02276_si_002.cif

## Accession Codes

CCDC 2099431–2099432 contain the supplementary crystallographic data for this paper. These data can be obtained free of charge via www.ccdc.cam.ac.uk/data_request/cif, or by emailing data_request@ccdc.cam.ac.uk, or by contacting The Cambridge Crystallographic Data Centre, 12 Union Road, Cambridge CB2 1EZ, UK; fax: +44 1223 336033.
