# Supplementary material for: Single-Crystal-to-Single-Crystal Cluster Transformation in a Microporous Molybdoarsenate(V)-Metalorganic Framework
Source: Inorg Chem. 2021 Sep 21;60(19):14913–23. doi: 10.1021/acs.inorgchem.1c02276 (PMC8493549; doi:10.1021/acs.inorgchem.1c02276)
Supplement: Supplementary file 1 — ic1c02276_si_001.pdf [file ic1c02276_si_001.pdf]

Single-Crystal-to-Single-Crystal Cluster Transformation in a Microporous Molybdoarsenate(V)-Metalorganic Framework

Nour Dissem,[†,Δ] Beñat Artetxe,*[,‡] Leire San Felices,[§] Garikoitz Beobide,[‡,‖] Oscar Castillo,[‡,‖] Estibaliz Ruiz-Bilbao,[‡] Luis Lezama,[‡] Maria dM. Vivanco,[π] Amor Haddad[⊥] and Juan M. Gutiérrez-Zorrilla*[,‡,‖]

[†] Laboratoire de Matériaux, Cristallochimie et Thermodynamique Appliquée, Faculté des Sciences de Tunis, Université de Tunis El Manar, 2092 Tunis, Tunisia

[‡] Departamento de Química Inorgánica, Facultad de Ciencia y Tecnología, Universidad del País Vasco UPV/EHU, P.O. Box 644, 48080 Bilbao, Spain

[§] Servicios Generales de Investigación SGIker, Facultad de Ciencia y Tecnología. Universidad del País Vasco UPV/EHU, P.O. Box 644, 48080 Bilbao, Spain

[⊥] Laboratoire des Matériaux et Cristallochimie, Institut Supérieur des Sciences Appliquées et Technologie, 5111 Mahdia, Tunisia

[π] Cancer Heterogeneity Lab, Center for Cooperative Research in Biosciences (CIC bioGUNE), Basque Research and Technology Alliance (BRTA), Bizkaia Technology Park, 48160 Derio, Spain

[‖] BCMaterials, Basque Center for Materials, Applications and Nanostructures, UPV/EHU Science Park, 48940 Leioa, Spain

Table of Contents

Figure S1. Top: FT–IR spectra of **1**. Bottom: Detail of the inorganic region....3

Figure S2. Molecular structures of the [*A*-$H_2As_2Mo_6O_{26}$]$^{(6-x)-}$, [*B*-$H_2As_2Mo_6O_{26}(H_2O)$]$^{(6-x)-}$ and [*B*'-$H_2As_2Mo_6O_{26}(H_2O)$]$^{(6-x)-}$ isomers....4

Figure S3. TGA/DTA curves for **1**....4

Figure S4. Identification of the final residue from the thermal decomposition of **1** by PXRD analyses....5

Figure S5. Variable-temperature powder X-ray diffraction (VT-PXRD) analyses on **1** from 30 to 600 °C, every 10 °C....6

Figure S6. ORTEP view of **1** with atom labelling (50% probability ellipsoids; hydrogen atoms and water molecules of hydration are omitted for clarity). Color code: Mo, white; As, green; Cu, purple; O, red; C, black; N, blue. Symmetry codes: i) –x, 1-y, 1-z; ii) -1+x, y, z....6

Table S1. Selected bond lengths (Å) for the {Cu(cyclam)}$^{2+}$ complexes in **1**, **2** and **2h**....7

Figure S7. Coordination geometries of the {Cu(cyclam)}$^{2+}$ complexes found in compounds **1**, **2** and **2h**, together with possible configurations of the cyclam ligand....7

Figure S8. a) View of the crystal packing of **1** along the crystallographic *y* axis. b) Projection of the crystallographic *yz* plane representing C–H···O-type contacts as dashed green lines. c) Surface representation of the solvent accessible channels running along the crystallographic *z* axis. Hydration water molecules are omitted for clarity....8

Table S2. Donor···Acceptor Distances (Å) for the O–H···O, N–H···O and C–H···O Interactions involving POM anions and metal-organic complexes in **1**, **2** and **2h**....9

Figure S9. TGA curve of **2** kept at room temperature in an open atmosphere for 24 h (**2h**)....9

Figure S10. Top: View of the crystal packing of **2** and **2h** along the crystallographic *x* axis. Bottom: detail of the different rotations of the POM anions (defined by the plane which contains its 6 Mo atoms) with respect to the *xy* plane....10

Figure S11. Variable-temperature X band EPR spectra of **1**....10

Figure S12. Fitting of the experimental EPR spectra of **1** for the X (using *Signal 1* for isolated centers, **A**; quasi-isotropic *Signal 2*, **B**; combination of both *Signals 1* and *2*, **C**) and Q (*Signal 1*, **D**) bands....11

Gas Sorption Properties. Experimental details....12

Figure S13. Models for the N_2 and CO_2 adsorbates. 12
Table S3. Lennard-Jones parameters and partial charges for the N_2 and CO_2 adsorbates. 12
Figure S14. a) Fragment of 3 used in the ESP charge calculation. The total charge of fragment was set to -6 e; b) Resulting ESP charges (q/e) upon the atoms of the structural models of the adsorbents. 13
Figure S15. PXRD pattern of **1** activated under vacuum at 70 °C for 24 h compared with that simulated from scXRD data for **2**. 13
Figure S16. Simulated adsorption isotherms for **2:** a) N_2 at 77 K; b) CO_2 at 273 K. 14
REFERENCES 14

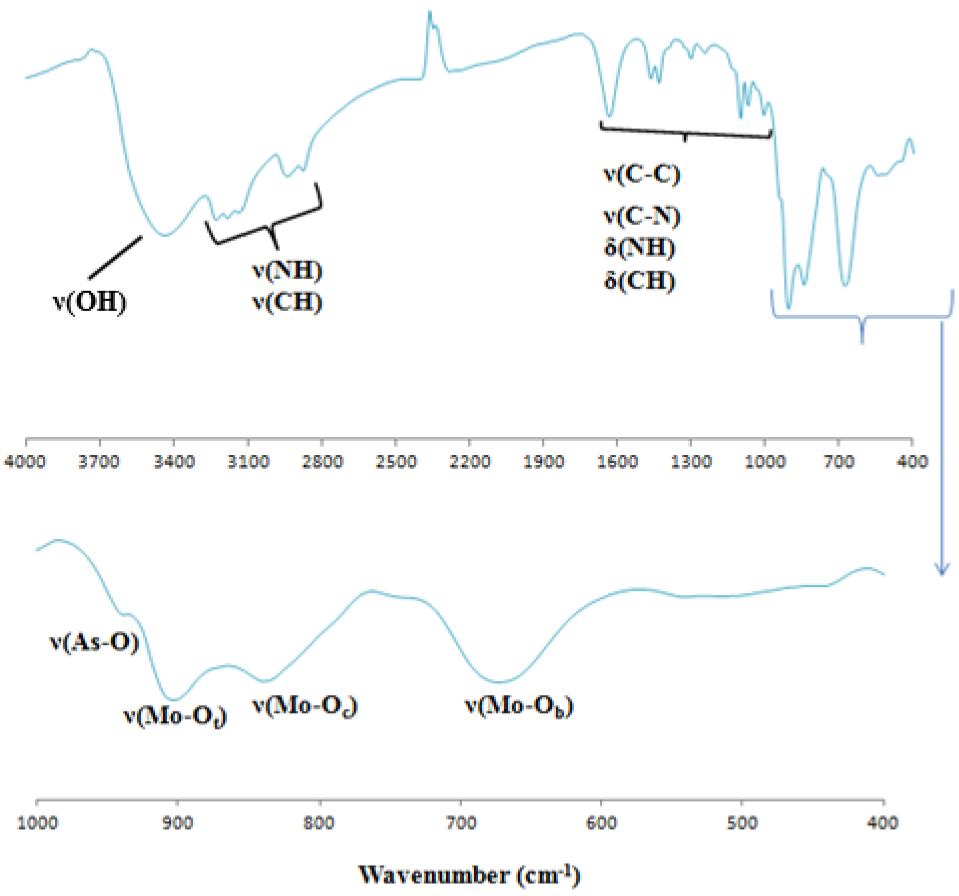

Figure S1. Top: FT–IR spectra of **1**. Bottom: Detail of the inorganic region.

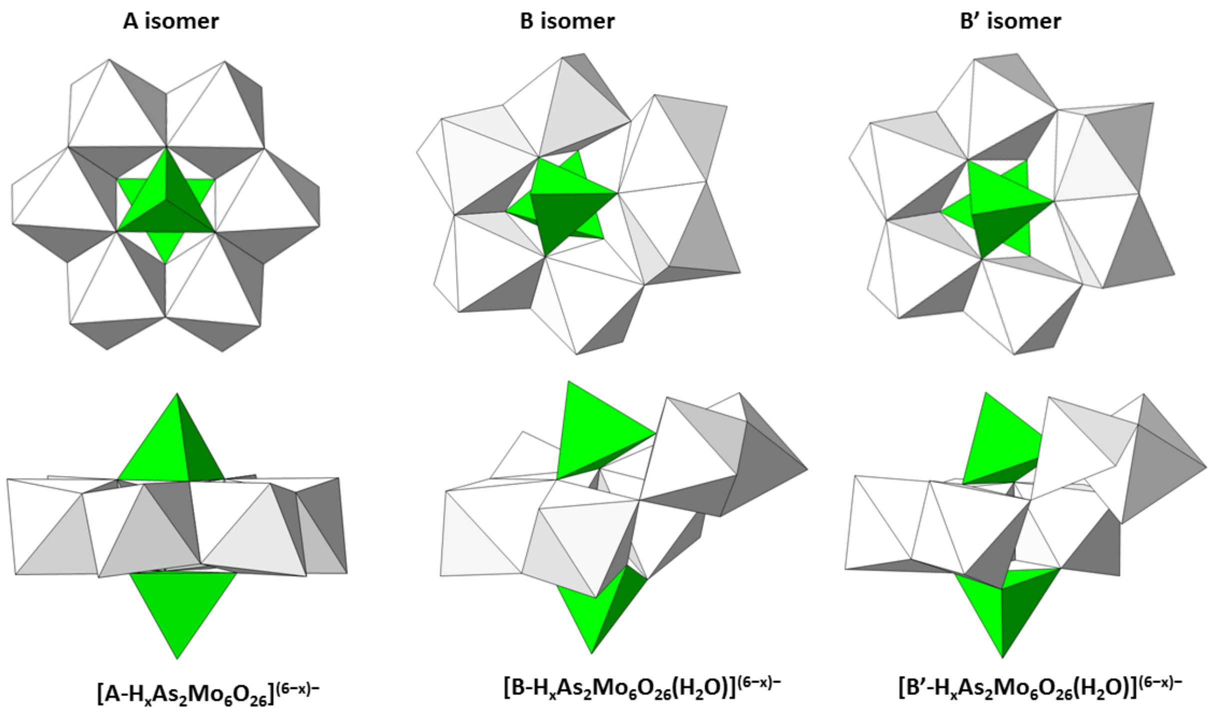

Figure S2. Molecular structures of the [*A*-$H_2As_2Mo_6O_{26}$]$^{(6-x)-}$, [*B*-$H_2As_2Mo_6O_{26}(H_2O)$]$^{(6-x)-}$ and [*B'*-$H_2As_2Mo_6O_{26}(H_2O)$]$^{(6-x)-}$ isomers.

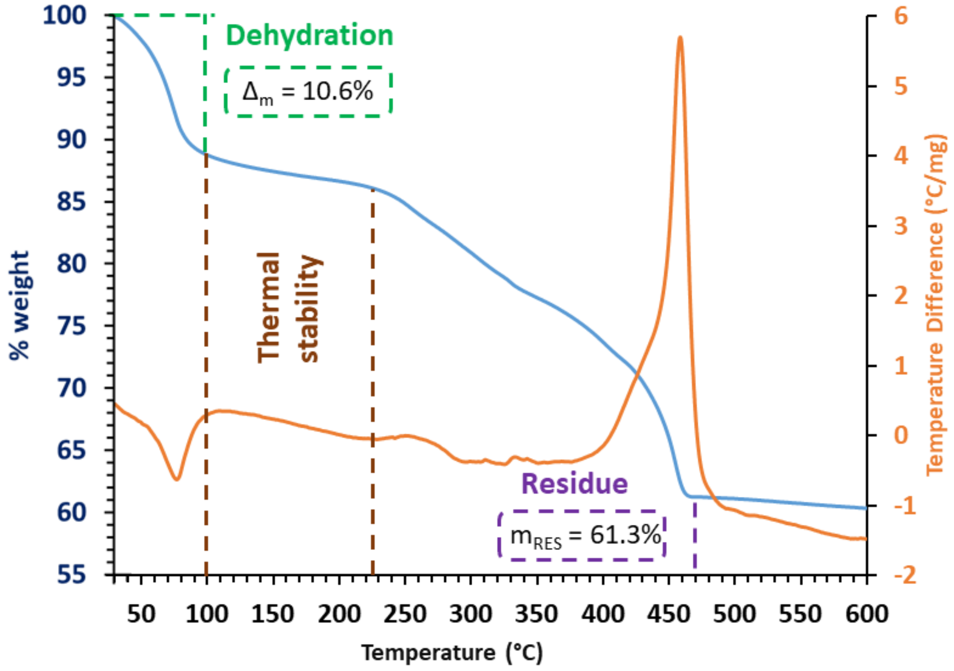

Figure S3. TGA/DTA curves for **1**.

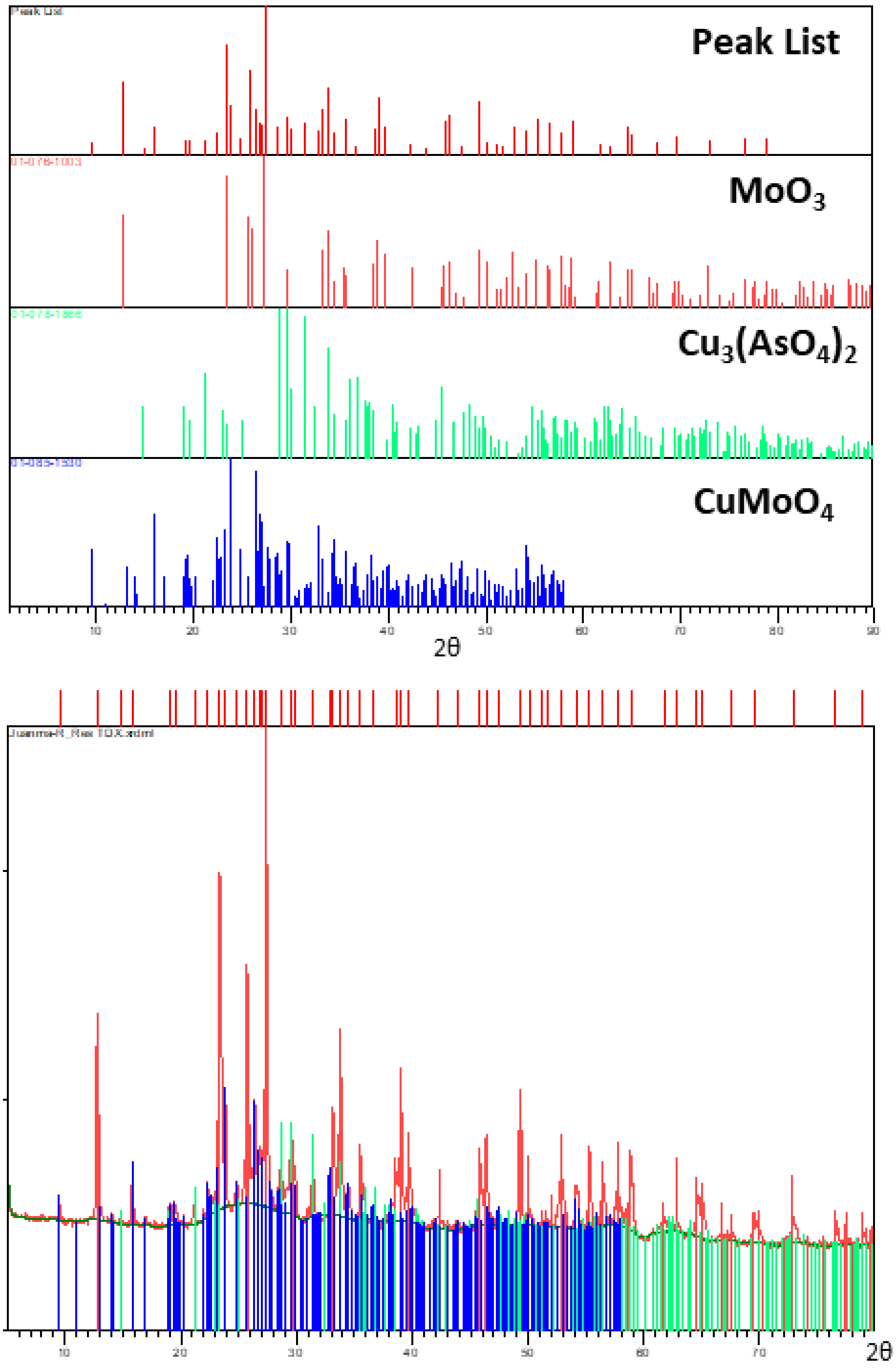

Figure S4. Identification of the final residue from the thermal decomposition of **1** by PXRD analyses (MoO_3, PDF: 01-076-1003: Kihlborg, L. *Ark. Kem.* **1963**, *21*, 357; $CuMoO_4$, PDF: 01-085-1530: Ehrenberg, H.; Weitzel, H.; Paulus, H.; Wiesmann, M.; Witschek, G.; Geselle, M.; Fuess, H. *J. Phys. Chem. Solids* **1997**, *58*, 153; $Cu_3(AsO_4)_2$, PDF: 01-078-1866: Effenberg, H. *Monatsh Chem.* **1988**, *119*, 1103)

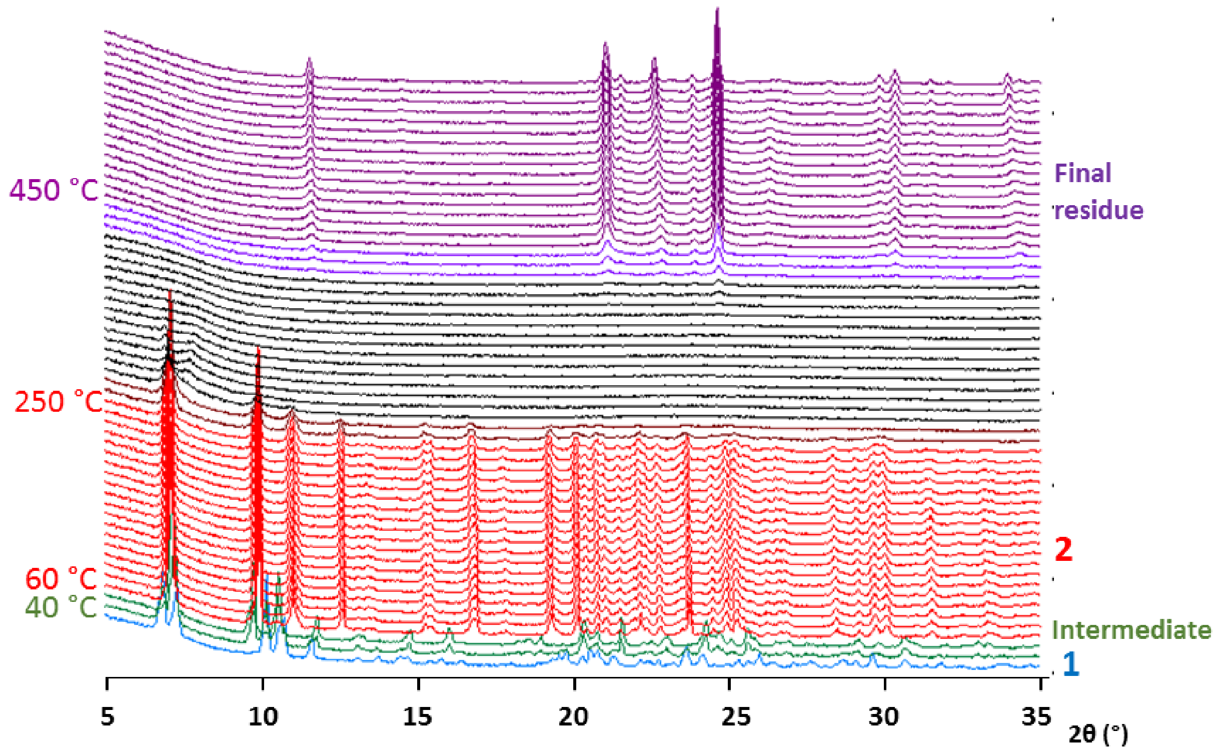

Figure S5. Variable-temperature powder X-ray diffraction (VT-PXRD) analyses on **1** from 30 to 600 °C, every 10 °C.

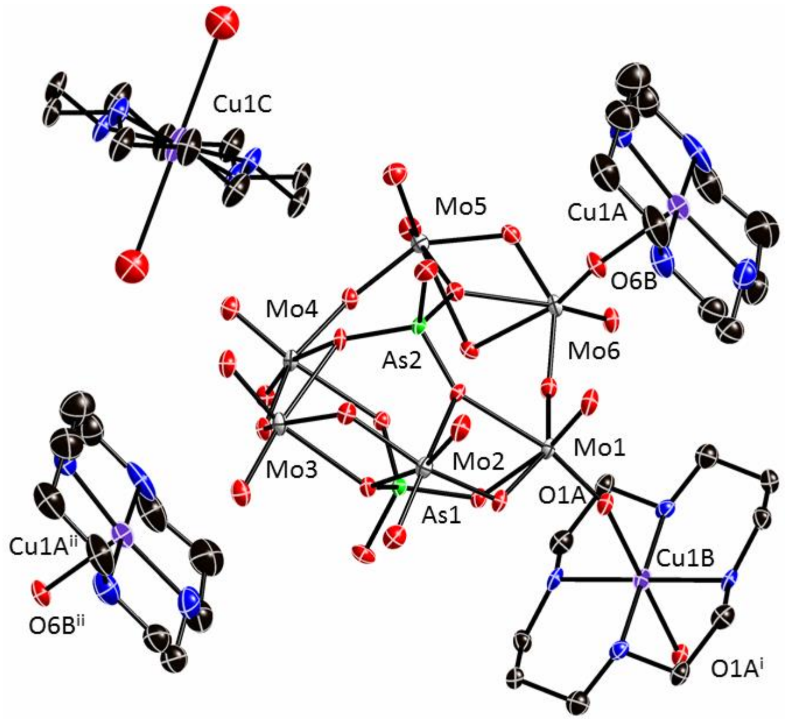

Figure S6. ORTEP view of **1** with atom labelling (50% probability ellipsoids; hydrogen atoms and water molecules of hydration are omitted for clarity). Color code: Mo, white; As, green; Cu, purple; O, red; C, black; N, blue. Symmetry codes: i) –x, 1-y, 1-z; ii) -1+x, y, z.

Table S1. Selected bond lengths (Å) for the $\{Cu(cyclam)\}^{2+}$ complexes in **1**, **2** and **2h**.

	**1**		**2**	**2h**
**Cu1A**		**Cu1A**		
Cu1A–N1A	2.024(7)	Cu1A–N1A	2.05(3)	1.98(4)
Cu1A–N4A	2.020(7)	Cu1A–N1A^{iii}	2.05(3)	1.98(4)
Cu1A–N8A	1.992(7)	Cu1A–N4A	1.93(3)	1.96(2)
Cu1A–N11A	2.016(7)	Cu1A–N4A^{iii}	1.93(3)	1.96(2)
Cu1A–O6B	2.321(5)	Cu1A–O3B	2.470(19)	2.50(3)
**Cu1B**		Cu1A–O3B^{iii}	2.470(19)	2.50(3)
Cu1B–N1B	2.017(6)	**Cu1B**		
Cu1B–N1B^{i}	2.017(6)	Cu1B–N1B	2.05(2)	2.03(2)
Cu1B–N4B	2.014(5)	Cu1B–N1B^{iv}	2.05(2)	2.03(2)
Cu1B–N4B^{i}	2.014(5)	Cu1B–N4B	1.96(2)	1.97(2)
Cu1B–O1A	2.439(4)	Cu1B–N4B^{iv}	1.96(2)	1.97(2)
Cu1B–O1A^{i}	2.439(4)	Cu1B–O1A	2.371(19)	2.38(3)
**Cu1C**		Cu1B–O1A^{iv}	2.371(19)	2.38(3)
Cu1C–N1C	1.994(6)			
Cu1C–N1C^{ii}	1.994(6)			
Cu1C–N4C	2.031(6)			
Cu1C–N4C^{ii}	2.031(6)			
Cu1C–O15W	2.705(7)			
Cu1C–O15W^{ii}	2.705(7)			

Symmetry codes: i) −x, 1−y, 1−z; ii) −x, −1−y, 2−z; iii) 1−x, 2−y, 1−z; iv) 1−x, 1−y, −z.

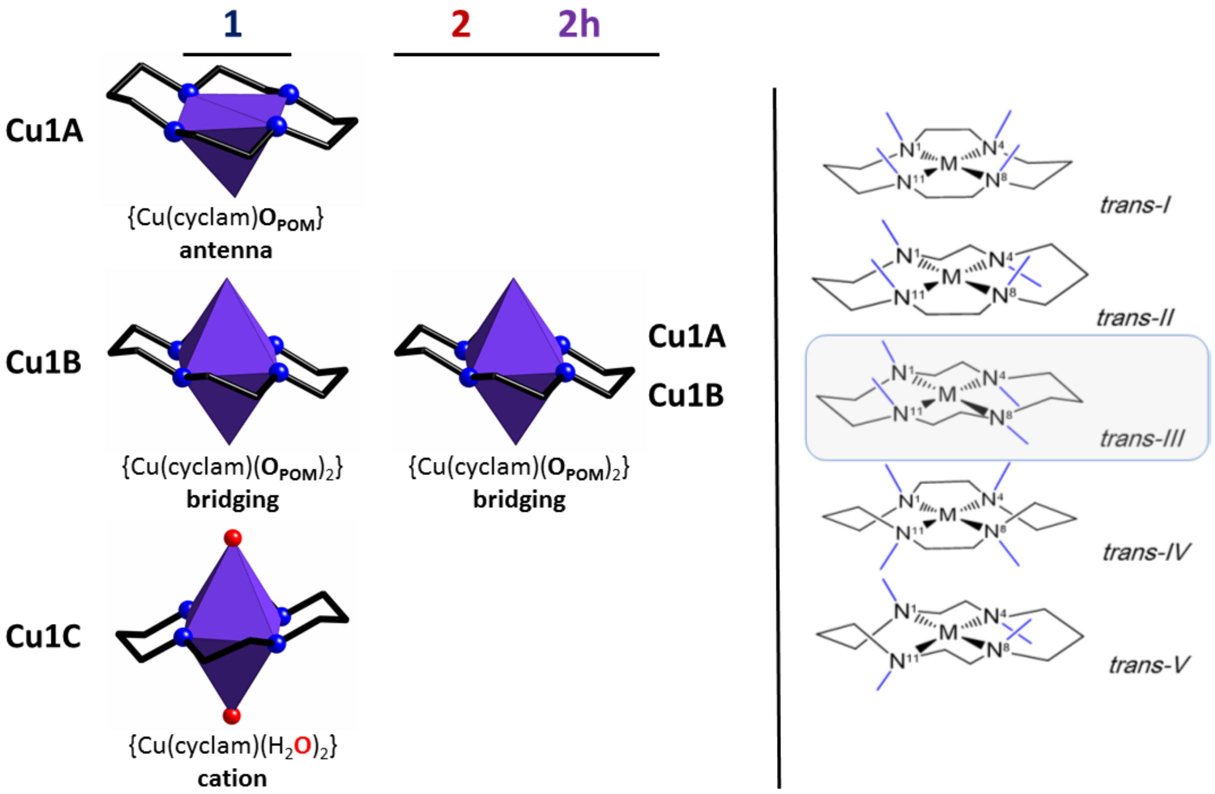

Figure S7. Coordination geometries of the $\{Cu(cyclam)\}^{2+}$ complexes found in compounds **1**, **2** and **2h**, together with possible configurations of the cyclam ligand.

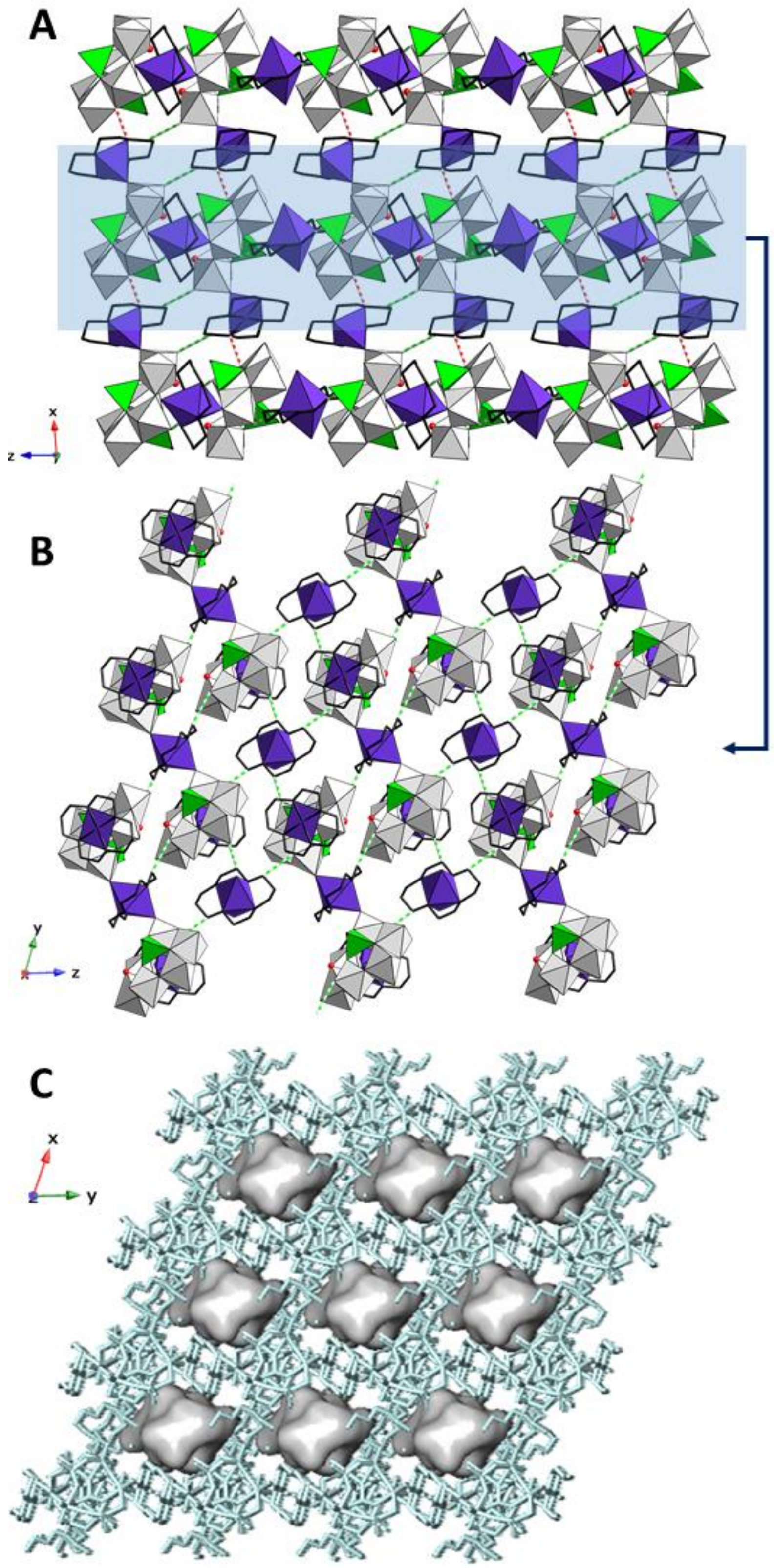

Figure S8. a) View of the crystal packing of **1** along the crystallographic *y* axis. b) Projection of the crystallographic *yz* plane representing C–H···O-type contacts as dashed green lines. c) Surface representation of the solvent accessible channels running along the crystallographic *z* axis. Hydration water molecules are omitted for clarity.

Table S2. Donor···Acceptor Distances (Å) for the O–H···O, N–H···O and C–H···O Interactions involving POM anions and metal-organic complexes in **1**, **2** and **2h**.

**1**		**2**		**2h**	
**Intramolecular**					
O1W–H1WA···O14X	2.689(7)				
**Intermolecular**					
POM···POM					
O1X–H1X···O5A^{i}	2.649(8)	O1X–H1X···O2B^{vi}	2.56(3)		
O1W–H1WB···O4A^{i}	2.702(7)				
POM···Cu1A					
N11A–H11A···O34^{iii}	2.920(11)	N1A–H1A···O1X	3.18(3)	N1A–H1A···O2A	3.15(4)
C12A–H12B···O6A^{iv}	3.082(12)	N5A–H5A···O13	3.00(4)		
		C3A···H3AA···O3A^{v}	3.42(5)		
POM···Cu1C		***POM···Cu1B***		***POM···Cu1B***	
O15W···O3B	2.832(8)	N4B–H4B···O12	3.10(3)	N1B–H1B···O12	3.06(3)
C2C–H2CA···O2X	3.025(10)	C2B–H2BA···O2B^{vii}	3.33(4)		
C7C–H7CA···O1B^{ii}	3.084(12)				

Symmetry codes: i) -*x*, –*y*, 1-*z*; ii) -*x*, –*y*, 2-*z*; iii) 1+*x*, *y*, *z*; iv) 1-*x*, -*y*, 1-*z*; v) -1+*x*, *y*, *z*; vi) –*x*, 1-*y*, 1-*z*; vii) –*x*, 1-*y*, -*z*

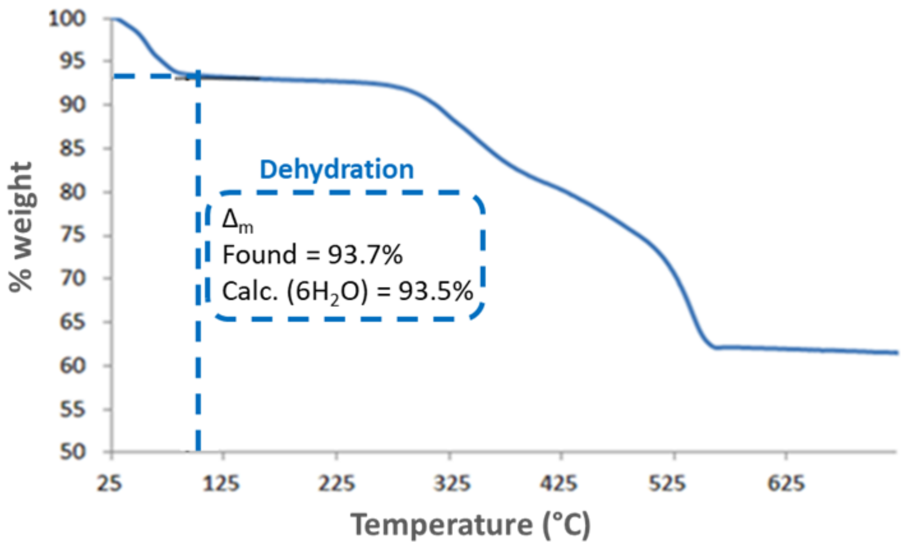

Figure S9. TGA curve of **2** kept at room temperature in an open atmosphere for 24 h (**2h**).

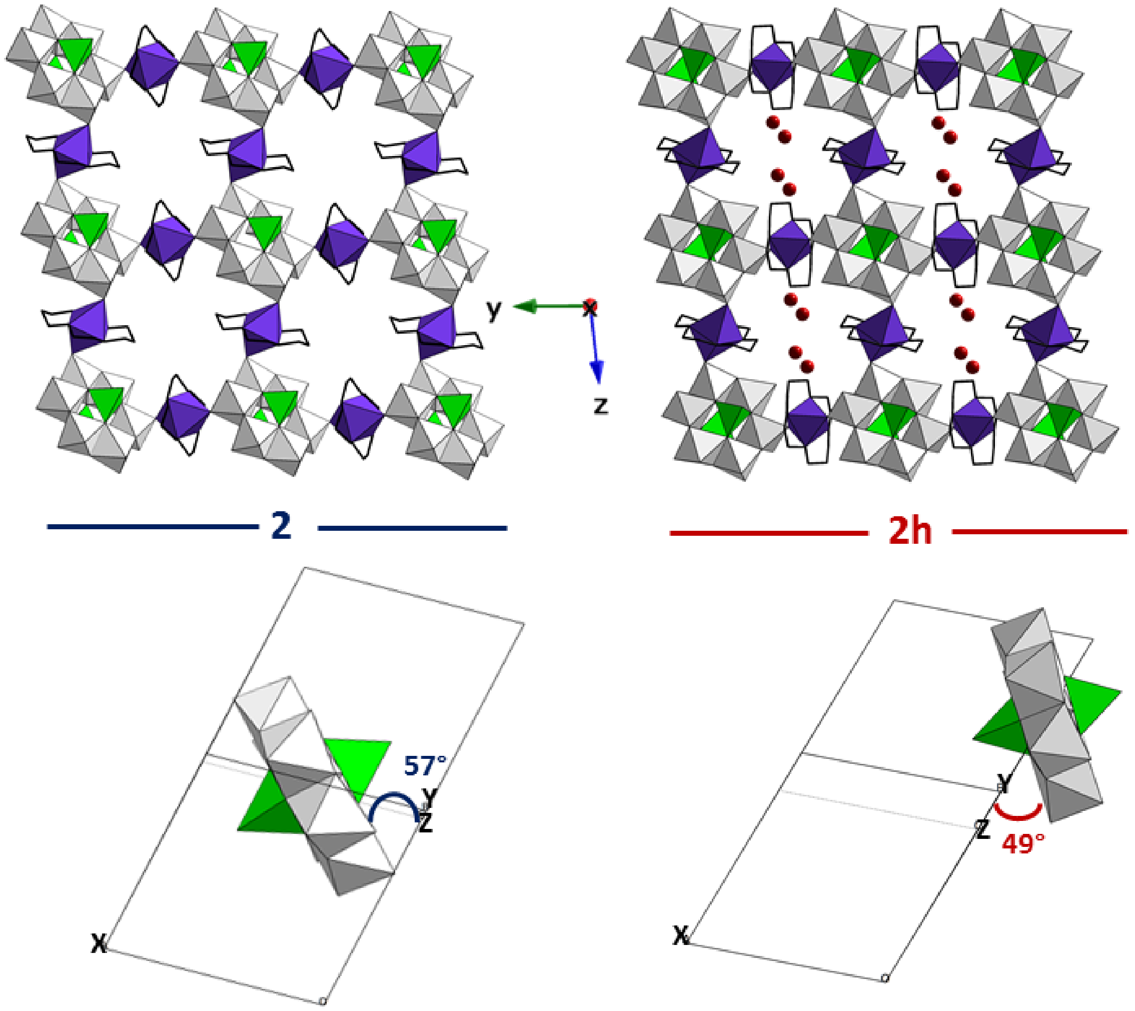

Figure S10. Top: View of the crystal packing of **2** and **2h** along the crystallographic *x* axis. Bottom: detail of the different rotations of the POM anions (defined by the plane which contains its 6 Mo atoms) with respect to the *xy* plane.

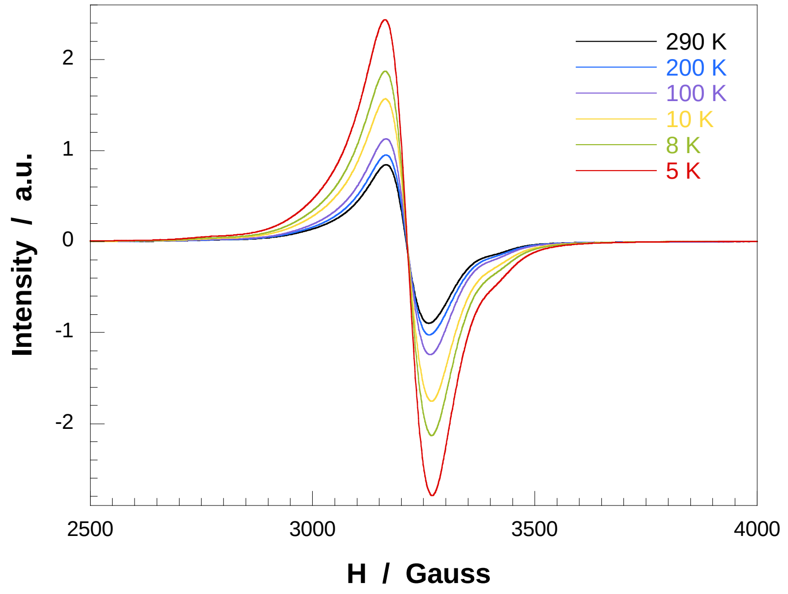

Figure S11. Variable-temperature X band EPR spectra of **1**.

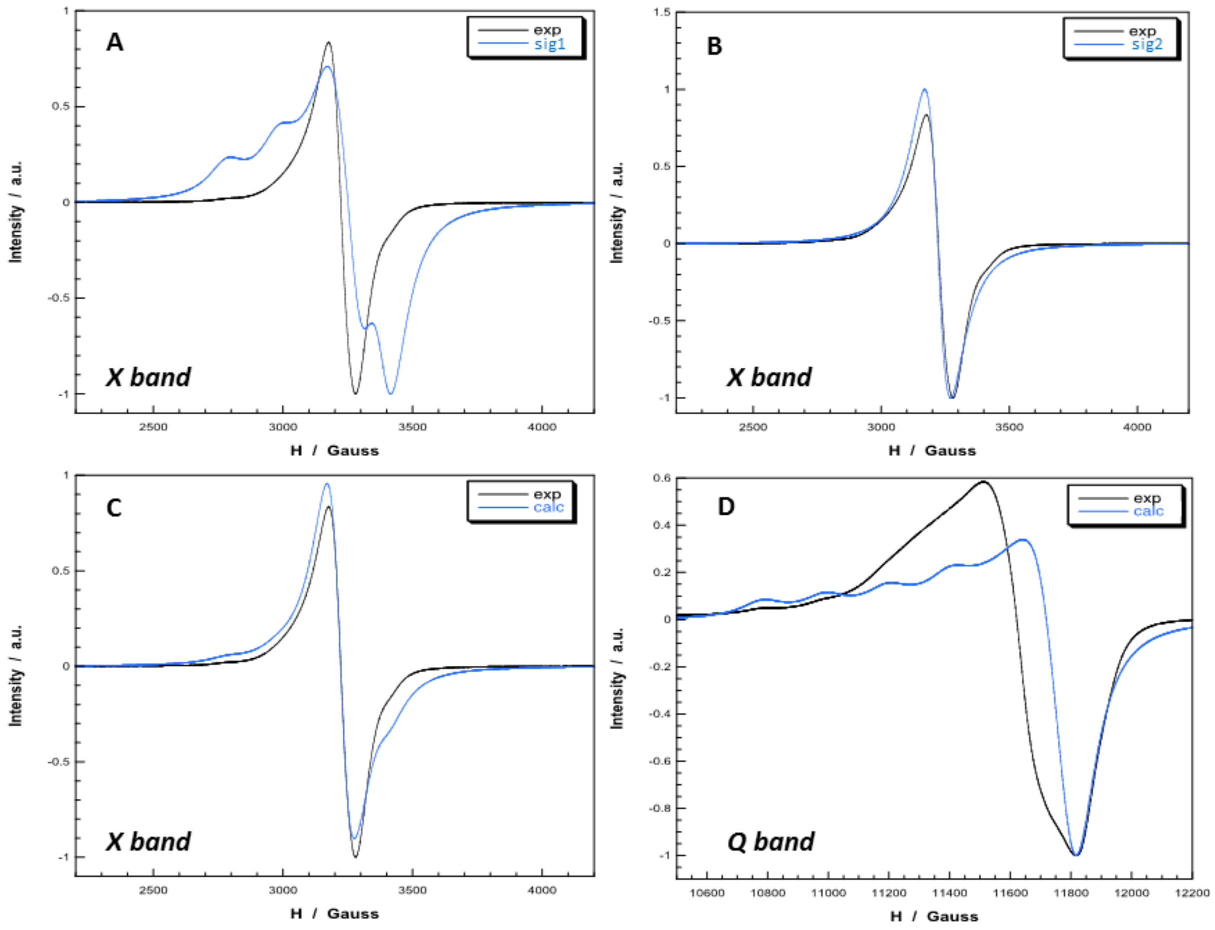

Figure S12. Fitting of the experimental EPR spectra of **1** for the X (using *Signal 1* for isolated centers, **A**; quasi-isotropic *Signal 2*, **B**; combination of both *Signals 1* and *2*, **C**) and Q (*Signal 1*, **D**) bands.

Gas Sorption Properties. Experimental details

Models for computational calculations

In all simulations, dispersive and electrostatic interactions were taken into account. Dispersive interactions were modeled using a Leonard-Jones (LJ) 12-6 potential. The parameters to represent the interaction between different atom types were calculated using Lorentz-Berthelot mixing rules. A cutoff radius of 12.5 Å was employed for dispersive interactions. Electrostatic interactions were modeled by assigning point charges to the atomic sites, and Ewald summation was used to account for the periodicity of the simulation box. The representation of the models and parameters used for the fluid molecules are gathered in Figure S12 and Table S3.

The models and parameters used to define the fluid molecules have been selected on the basis of previous studies. For the N_2 molecule, the LJ parameters were taken from the TraPPE model.[1] This model simulates the quadrupolar moment of N_2 by placing two negative charges (−0.482) in the positions of the N atoms and a positive charge in the center of mass (+0.964). The LJ parameters used to represent the CO_2 interactions were taken from the work by García-Sánchez et al,[2] and consist of a modified version of the TraPPE potential model. The LJ parameters for all of the atoms of the adsorbent (**2**) were taken from the universal force field (UFF).[3] The partial charges to represent the electrostatic potential inside the pores were derived from DFT calculations using the ESP method as described by Singh and Kollman,[4] which is implemented in the DMOL3 code.[5] The DNP basis set and the PBE exchange-correlation functional were selected for this calculation.[6] Due to the large number of atoms that are contained in the unit cell of **2**, the charges were averaged considering the atom type prior to set the calculated ESP charges into the adsorbent model (Figure S13).

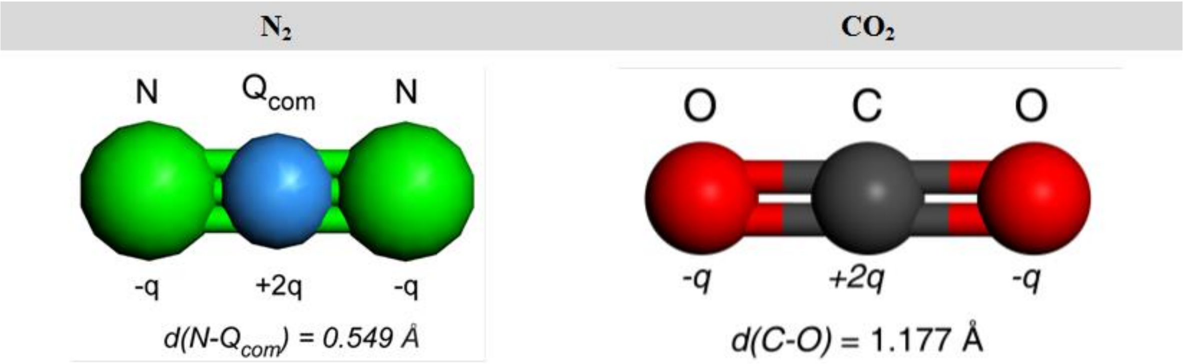

Figure S13. Models for the N_2 and CO_2 adsorbates.

Table S3. Lennard-Jones parameters and partial charges for the N_2 and CO_2 adsorbates.

	r_0 / Å	D_0 / kJ mol^{-1}	q / e
N_2			
N	3.7153	0.2993	–0.482
Q_{com}	--	--	+0.964
CO_2			
C	3.0811	0.2444	+0.5810
O	3.3865	0.7121	–0.2905

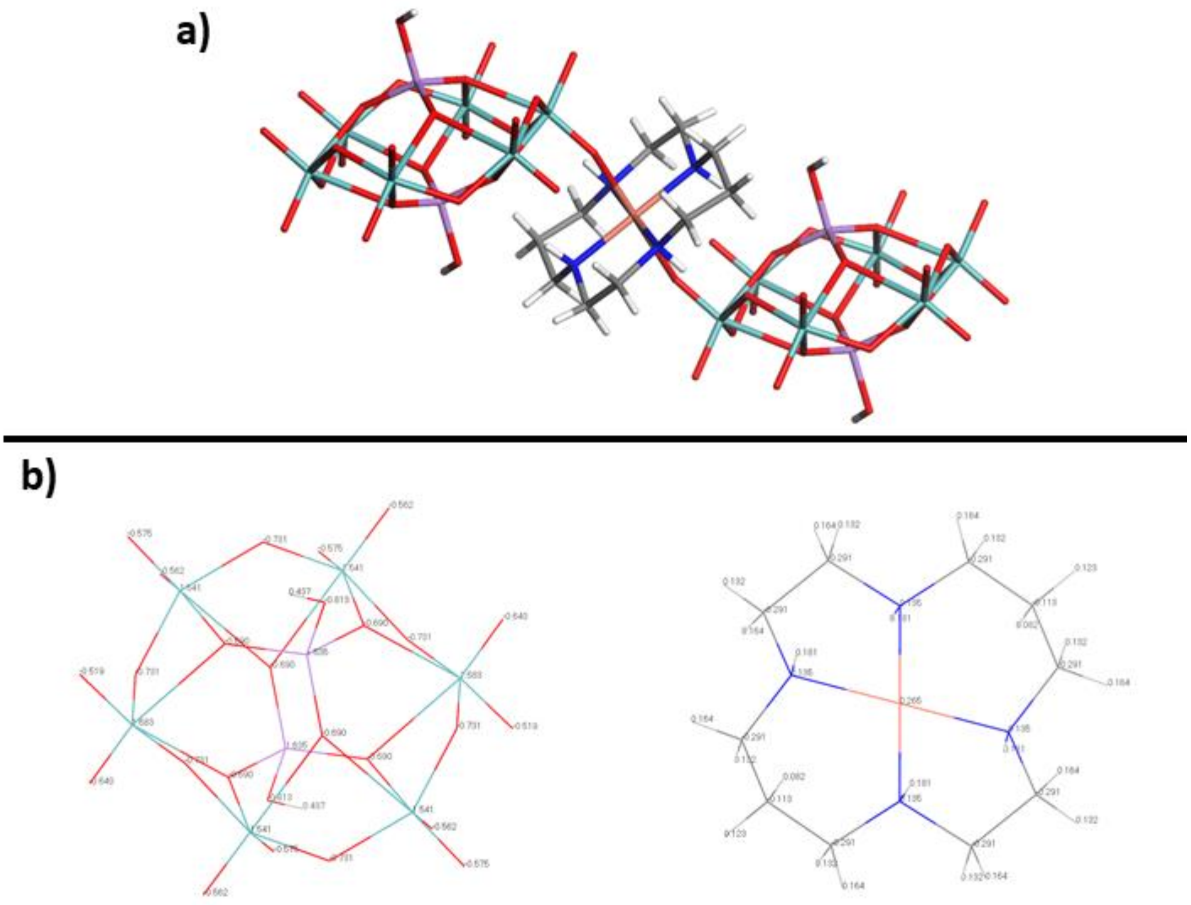

Figure S14. a) Fragment of 3 used in the ESP charge calculation. The total charge of fragment was set to -6 e; b) Resulting ESP charges (q/e) upon the atoms of the structural models of the adsorbents.

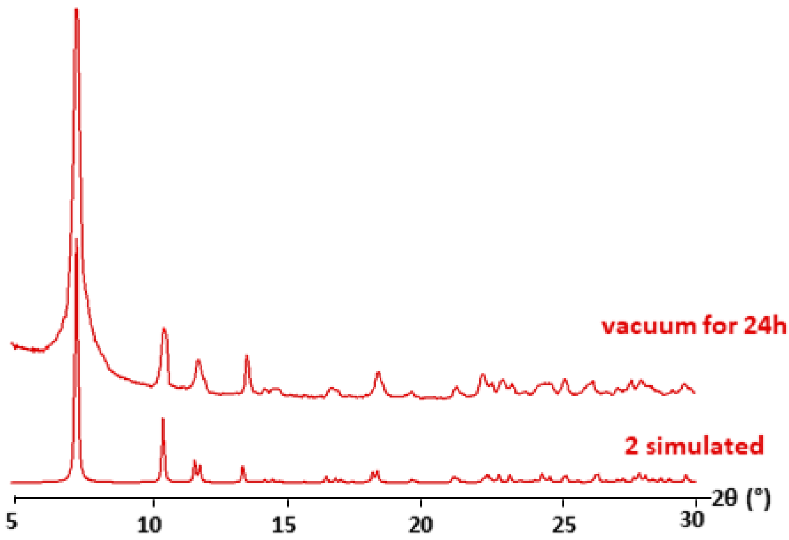

Figure S15. PXRD pattern of **1** activated under vacuum at 70 °C for 24 h compared with that simulated from scXRD data for **2**.

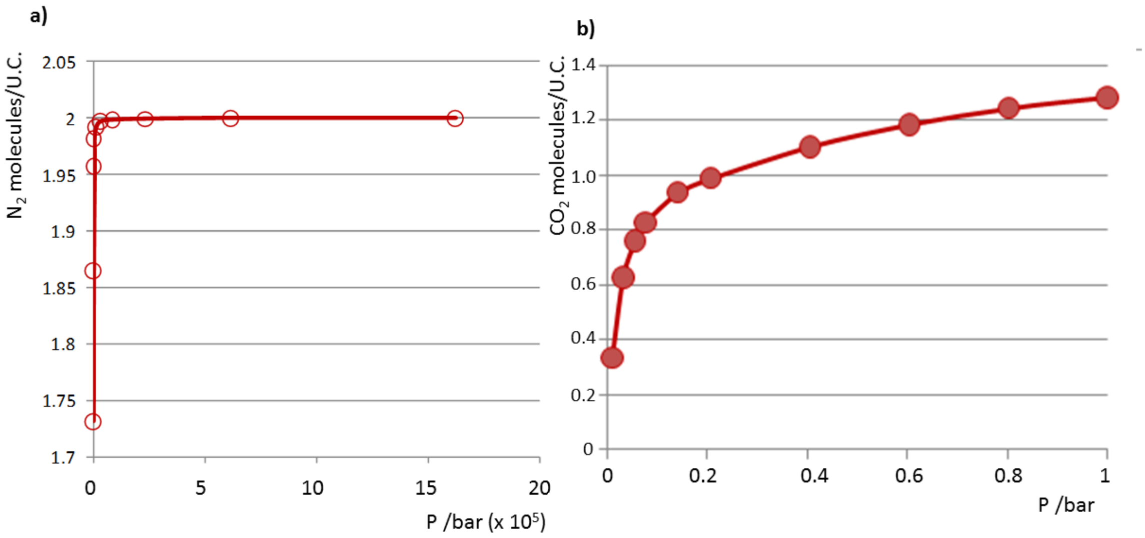

Figure S16. Simulated adsorption isotherms for **2:** a) N_2 at 77 K; b) CO_2 at 273 K.

REFERENCES

[1] Potoff, J. J.; Siepmann, J. I. Vapor–liquid equilibria of mixtures containing alkanes, carbon dioxide, and nitrogen. *AIChE J.* **2001**, *47*, 1676–1682.

[2] García-Sánchez, A.; Ania, C. O.; Parra, J. B.; Dubbeldam, D.; Vlugt, T. J. H.; Krishna, R.; Calero, S. Transferable Force Field for Carbon Dioxide Adsorption in Zeolites. *J. Phys. Chem. C* **2009**, *113*, 8814–8820.

[3] Rappe, A. K.; Casewit, C. J.; Colwell, K. S.; Goddard III, W. A.; Skiff, W. M. UFF, a full periodic table force field for molecular mechanics and molecular dynamics simulations, *J. Am. Chem. Soc.* **1992**, *114*, 10024–10035.

[4] Singh, U. C.; Kollman, P. A. An approach to computing electrostatic charges for molecules. *J. Comput. Chem.* **1984**, *5*, 129–145.

[5] Delley, B. From molecules to solids with the $DMol^3$ approach. *J. Chem. Phys.* **2000**, *113*, 7756–7764.

[6] Perdew, J. P.; Burke, K.; Ernzerhof, M. Generalized Gradient Approximation Made Simple. *Phys. Rev. Lett.* **1996**, *77*, 3865–3868.
